# The role of PD-1/PD-L1 axis in idiopathic pulmonary fibrosis: Friend or foe?

**DOI:** 10.3389/fimmu.2022.1022228

**Published:** 2022-12-05

**Authors:** Aimin Jiang, Na Liu, Jingjing Wang, Xiaoqiang Zheng, Mengdi Ren, Wei Zhang, Yu Yao

**Affiliations:** ^1^ Department of Medical Oncology, The First Affiliated Hospital of Xi’an Jiaotong University, Xi’an, China; ^2^ Institute for Stem Cell & Regenerative Medicine, The Second Affiliated Hospital of Xi’an Jiaotong University, Xi’an, China; ^3^ Military Physical Education Teaching and Research Section of Air Force Medical Service Training Base, Air Force Medical University, Xi’an, China

**Keywords:** PD-1, PD-L1, idiopathic pulmonary fibrosis, profibrotic role, immunomodulatory role, therapeutic target

## Abstract

Idiopathic pulmonary fibrosis (IPF) is a devastating interstitial lung disease with a bleak prognosis. Mounting evidence suggests that IPF shares bio-molecular similarities with lung cancer. Given the deep understanding of the programmed cell death-1 (PD-1)/programmed death-ligand 1 (PD-L1) pathway in cancer immunity and the successful application of immune checkpoint inhibitors (ICIs) in lung cancer, recent studies have noticed the role of the PD-1/PD-L1 axis in IPF. However, the conclusions are ambiguous, and the latent mechanisms remain unclear. In this review, we will summarize the role of the PD-1/PD-L1 axis in IPF based on current murine models and clinical studies. We found that the PD-1/PD-L1 pathway plays a more predominant profibrotic role than its immunomodulatory role in IPF by interacting with multiple cell types and pathways. Most preclinical studies also indicated that blockade of the PD-1/PD-L1 pathway could attenuate the severity of pulmonary fibrosis in mice models. This review will bring significant insights into understanding the role of the PD-1/PD-L1 pathway in IPF and identifying new therapeutic targets.

## 1 Introduction

Idiopathic pulmonary fibrosis (IPF) is a chronic progressive fibrotic interstitial lung disease characterized by chronic epithelial injury and exhausted repair capacity of the alveolar compartment (1, 2). IPF occurs mainly in individuals over 60, with progressive dyspnea and irreversible pulmonary function damage being the most prevalent manifestations (1). It is estimated that the prevalence of IPF is staggering at 10-20 per 100,000 people in Western countries (3). Currently, although some newly authorized therapeutic drugs such as Pirfenidone and Nintedanib are approved for IPF treatment (4–6), they only moderately attenuate lung function damage and could not reverse disease progression or reduce case-fatality (3). Hence, IPF is still a lethal “silent killer” and novel treatment strategies are urgently needed to alleviate its impact on patients’ lives.

It is well documented that interstitial lung diseases correlate with lung cancer development. Indeed, IPF patients have a fivefold increased risk of lung cancer development compared with ordinary people (7). Besides, accumulating studies have demonstrated that pre-existing IPF hurts the prognosis of lung cancer patients regardless of anticancer modalities (8, 9). Meanwhile, numerous studies also investigated the association between IPF and lung cancer. For example, in a review, Tzouvelekis et al. summarized the common pathogenic mechanisms between IPF and lung cancer, elucidating that they share many pathogenic similarities, including genetic and epigenetic markers (7). Gaining insight into common molecular characteristics between IPF and lung cancer could provide more treatment options for these patients.

The immune homeostasis maintenance role of the programmed cell death-1 (PD-1)/programmed death-ligand 1 (PD-L1) pathway in human diseases is well elucidated in recent studies. On the one hand, activation of the PD-1/PD-L1 signaling pathway could regulate the intensity of immune response in peripheral tissues and maintain immune tolerance to self-antigens by inhibiting T cells hyperactivation and cytokines secretion (such as IL-10 and IFN-γ) (10). However, on the other hand, in the tumor microenvironment (TME), the PD-1/PD-L1 axis is also employed by cancer cells to escape immunological surveillance (11). TCR signaling upregulates the expression of PD-1 on the T cell surface, which binds to PD-L1 on cancer cells to exert negative regulatory effects and thus impair the antitumor function of T cells (12). Therefore, inhibiting the PD-1/PD-L1 axis has been successfully used to reverse the immunosuppressive TME, thereby restoring the normal antitumor effect of T cells. Given this advantage, immune checkpoint inhibitors (ICIs) targeting the PD-1/PD-L1 axis have shown promising antitumor effects in various malignancies (11).

Existing evidence identified that aside from cancer cells, PD-L1 is also expressed on some types of immune cells (11) and stromal cells (13–16). Interestingly, recent studies have shown that PD-L1 is aberrantly expressed on human and mouse lung fibroblasts. Given the increased recognition of the similarities between lung cancer and IPF, more and more studies focused on the role of the PD-1/PD-L1 axis in IPF and investigated the potential therapeutic effect of PD-1/PD-L1 blockades on IPF. Nevertheless, the conclusions are equivocal and part of the mechanism remains unclear. In this review, we will summarize the role of the PD-1/PD-L1 axis in IPF based on clinical studies and animal models.

## 2 PD-1/PD-L1 axis and IPF in clinical studies

Many studies have investigated the relationship between the PD-1/PD-L1 axis and IPF in human samples, and **Table 1** summarizes their main findings. Although different methods are applied to measure PD-1 or PD-L1 expression levels, most studies have identified abnormal PD-1 or PD-L1 expression in the lung tissue or peripheral blood samples from IPF patients compared with healthy individuals. Regarding the detailed detecting methods, immunohistochemistry (IHC) was most commonly used to evaluate the expression levels of PD-1 and PD-L1 in the lung samples from IPF patients (19, 22, 23, 25). For instance, Kronborg-White and colleagues detected the cellular membrane PD-L1 (mPD-L1) expression in the lung specimen from IPF patients using the DAKO PD-L1 IHC 22C3 PharmDx Kit. They found positive expression of mPD-L1 in alveolar and/or bronchiolar epithelial cells in the lung tissue of most IPF patients (19). However, in a small sample size study, Jovanovic et al. found that PD-L1 was negatively expressed in fibroblasts and myofibroblasts in lung tissue from IPF patients by IHC staining (23). Intriguingly, they observed that mPD-L1 is overexpressed on alveolar macrophages (23). One study applied IHC to detect the PD-1 expression levels in lung tissue from healthy donors, IPF patients, and lung cancer patients. The results suggested that PD-1 was significantly overexpressed in IPF and lung cancer samples compared to healthy donors (22). Further, Habiel identified a higher infiltration level of CD8^+^ cytotoxic CD28^null^ T cells in the lung tissue of IPF patients, and these T cells expressed higher levels of PD-1 (26). Several studies also used immunofluorescence (IF) (17, 27), flow cytometry (FCM) (25–27), Western blot (WB) (27), and mass cytometry (20) to explore the expression pattern of PD-1/PD-L1 axis in IPF patients. Interestingly, some studies explored the expression levels of PD-1 and PD-L1 in the peripheral blood of IPF patients and healthy individuals (18, 22, 23). Exploiting FCM, Wang et al. revealed that PD-1 and PD-L1 were overexpressed on the CD4^+^ T cells of IPF patients’ peripheral blood (18). Similarly, Ni et al. also indicated that the percentage of PD-1^+^ lymphocytes in the peripheral blood of IPF patients was also elevated (22). However, there was no significant elevation of PD-L1 expression level in the peripheral blood leukocytes of IPF patients compared with healthy donors (22). Furthermore, there were two small sample size studies detected the soluble PD-L1 (sPD-L1) plasma concentration in IPF patients (23, 24). Jovanovic et al. applied a sandwich enzyme-linked immunosorbent assay (ELISA) kit to detect the sPD-L1 plasma concentration in 23 IPF patients who did not undergo surgical biopsy (23). They identified that the serum sPD-L1 concentration of IPF patients was significantly higher compared with healthy donors (*P*<0.01) (23). Besides, another study also observed higher level of sPD-L1 in patients with IPF compared to healthy individuals using ELISA (24). Taken together, these findings suggest abnormal expression of the PD-1/PD-L1 axis in IPF patients. The PD-1/PD-L1 pathway may contribute to the occurrence and development of IPF. However, well-designed and prospective studies should be conducted to make it clear since all currently published studies are retrospectively designed and have a small sample size.

**Table 1:** The expression level of the PD-1/PD-L1 in human studies.

First author	PD-1/PD-L1	Patients	Sample	Method	Cell types	Expression level	Ref.
Xia Guo	PD-L1	IPF/normal	Lung	IF	/	Upregulation	(17)
Bing Wang	PD-1	IPF/normal	Peripheral blood	FCM	CD4^+^ T cells	Upregulation	(18)
	PD-L1	IPF/normal				Upregulation	
Sissel Kronborg-White	mPD-L1	IPF/normal	Lung	IHC	Alveolar and/or bronchiolar epithelial cells	Upregulation	(19)
Lu Cui	PD-L1	IPF/normal	Lung	Mass cytometry	Fibroblasts	Upregulation	(20)
Yan Geng	PD-L1	IPF/normal	Lung	IF WB FCM	Fibroblasts	Upregulation	(21)
Ke Ni	PD-1	IPF/normal	Lung	IHC	/	Upregulation	(22)
			Peripheral blood	FCM	T lymphocytes	Upregulation	
	PD-L1		Peripheral blood	FCM	T lymphocytes	No change	
Dragana Jovanovic	mPD-L1 sPD-L1	IPF/normal	Lung	IHC	Alveolar macrophages	Upregulation	(23)
			Plasma	ELISA	/	Elevation	
M Roksandic Milenkovic	sPD-L1	IPF/normal	Plasma	ELISA	/	Elevation	(24)
Lindsay J. Celada	PD-1	IPF/normal	Lung	FCM	Th17 cells	Upregulation	(25)
				IHC	/	Upregulation	
	PD-L1			IHC	/	Upregulation	
David M. Habiel	PD-1	IPF/normal	Lung	FCM	CD8^+^ cytotoxic CD28^null^ T cells	Upregulation	(26)

IPF, idiopathic pulmonary fibrosis; PD-1, programmed cell death 1; PD-L1, programmed death-ligand 1; IF, immunofluorescence; FCM, flow cytometry; mPD-L1, membrane programmed death-ligand 1; sPD-L1, soluble programmed death-ligand 1; IHC, immunohistochemistry; WB, western blot; ELISA, enzyme linked immunosorbent assay; Th17, T helper 17.

## 3 PD-1/PD-L1 axis and IPF in animal models

Numerous studies have investigated the expression pattern and role of the PD-1/PD-L1 axis in IPF through animal models. In this part, we will only discuss the expression disorder of the PD-1/PD-L1 axis in IPF animal models, and its roles in IPF will be summarized in the next section. **Table 2** depicts current studies focusing on the expression pattern of the PD-1/PD-L1 axis in pulmonary fibrosis *via* animal models. The intratracheal administration of the cytotoxic drug bleomycin to C57BL/6 mice is known to be the most commonly used murine model to simulate the pathological process of human IPF (31). Bleomycin contributes to fibrosis by inducing alveolar epithelial injury *via* DNA cleavage, free radical formation, and deoxynucleotide oxidizing reaction (32). Even though it could not fully reflect the clinical characteristics and pathological process of human lung fibrosis, it remains crucial in preclinical studies of anti-fibrotic compounds screening and pulmonary fibrosis mechanisms investigation (31). As mentioned before, PD-L1 is not only expressed on tumor cells, but can also be detected on some types of immune cells (11) and stromal cells (13–16). Emerging evidence has identified that PD-L1 is also expressed on fibroblasts/myofibroblasts (17, 20, 21, 29) and mesenchymal stem cells (MSCs) (22) in the lung from IPF patients or murine models. In a bleomycin-induced mouse pulmonary fibrosis model, Lu and colleagues observed that the expression level of PD-L1 is significantly upregulated in the lung tissue (29). Besides, they further investigated the protein expression level of PD-L1 in primary mouse lung fibroblasts to confirm PD-L1 upregulation at the cellular level. As expected, the primary mouse lung fibroblasts expressed a higher level of PD-L1 (29). Consistent results were also reported in other studies (17, 20). As a binding molecule of PD-L1 on T cells, several studies have also detected the expression pattern of PD-1 in the bleomycin-induced pulmonary fibrosis murine model (25, 30). Wang et al. observed that PD-1 was significantly upregulated in the lung tissue of the pulmonary fibrosis mice model *via* IHC staining. Besides, they found that PD-1 was also overexpressed on CD4^+^ T cells of peripheral blood (25, 30). Nevertheless, they failed to quantify the PD-1 expression on T cells in the lung tissue. Celada and colleagues revealed that PD-1 was positively expressed on CD4^+^ T cells in the mice lung specimen (25). In addition, Cui et al. found that PD-1 was upregulated on CD8^+^T cells in the lung of the bleomycin chemical injury murine model, indicating an immunosuppressive environment existed in lung fibrosis (20).

**Table 2:** The expression level of the PD-1/PD-L1 in animal models.

First author	PD-1/PD-L1	Animal	Exposure	Sample	Cell types	Method	Expression level	Ref.
Youliang Zhao	PD-1	6-8 weeks male C57BL/6 mice	Silica	Lung	/	WB IHC FCM PCR	Upregulation	(28)
	PD-L1			Lung	/		Upregulation	
Ye Lu	PD-L1	4-6 weeks female C57BL/6 mice	BLM	Lung	Fibroblasts	WB IHC	Upregulation	(29)
Xia Guo	PD-L1	12-16 weeks male C57BL/6 mice	BLM	Lung	Fibroblasts	IF	Upregulation	(17)
Dong Wang	PD-1	8 weeks male C57BL/6 mice	BLM	Lung	/	IHC	Upregulation	(30)
				Peripheral blood	CD4^+^ T cells	FCM	Upregulation	
Lu Cui	PD-L1	11-12 weeks male C57BL/6 mice	BLM	Lung	Fibroblasts	Mass cytometry IF FCM	Upregulation	(20)
		JUN-induced lung fibrosis mice	JUN	Lung	Fibroblasts		Upregulation	
		IL-6 knock out mice and wildtype	BLM	Lung	Fibroblasts		Upregulation	
		NSG mice	Primary human fibrotic lung fibroblasts engraftation	Lung	Fibroblasts		Upregulation	
	PD-1	11-12 weeks male C57BL/6 mice	BLM	Lung	CD8^+^T cells	Mass cytometry	Upregulation	
Yan Geng	PD-L1	6-8 weeks female NSG mice	CD274^high^ and CD274^low^ IPF lung normal fibroblasts injection	Lung	Fibroblasts	PCR RNA-seq	Upregulation	(21)
Ke Ni	PD-L1	4-6 weeks Rag2^-/-^γc^-/-^mice	BLM	Lung	MSC	FCM	Upregulation	(22)
Lindsay J. Celada	PD-1	7-9 weeks old C57BL/6J mice	BLM	Lung	CD4^+^ T cells	FCM	Upregulation	(25)

IPF, idiopathic pulmonary fibrosis; PD-1, programmed cell death 1; PD-L1, programmed death-ligand 1; IF, immunofluorescence; FCM, flow cytometry; IHC, immunohistochemistry; WB, western blot; PCR, polymerase chain reaction; BLM, bleomycin; NSG, NOD-SCID-IL2Rγc^–/–^; RNA-seq, RNA sequencing; MSC, mesenchymal stem cell.

In recent years, immunodeficient mice have become increasingly useful as preclinical animal models for studying human diseases since they are also capable of engrafting human tissues (21, 33–36). Some studies also employed the humanized murine model to investigate the expression pattern and role of the PD-1/PD-L1 axis in IPF (20–22, 26). Geng and colleagues established a humanized murine model of pulmonary fibrosis through intravenous injection of invasive IPF lung fibroblasts into NOD-SCID-IL2Rγc^–/–^ (NSG) mice (21). More severe pulmonary fibrosis was observed in the lung of mice injected with invasive lung fibroblasts than in mice injected with noninvasive IPF lung fibroblasts. By performing RNA sequencing (RNA-*seq*) analysis and experimental validation, they observed that PD-L1 is significantly upregulated on the invasive fibroblasts of the pulmonary fibrosis mice model. Besides, in a humanized mouse model in which the researchers successfully engrafted primary human fibrotic lung fibroblasts in NSG mice underneath the kidney capsule, upregulation of PD-L1 was also observed on the fibroblasts in the lung (20).

Genetically modified and silica-induced lung fibrosis murine models were also applied to investigate the expression pattern and role of the PD-1/PD-L1 pathway in pulmonary fibrosis. Consistent with the PD-L1 expression level in the lung tissue of the bleomycin-driven pulmonary fibrosis murine model, Cui and colleagues observed that PD-L1 was also positively expressed in the lung of IL-6 knockout mice (20). In a silica-induced pulmonary fibrosis mice model, the researchers adopted multiple assays to evaluate the changes in the mRNA and protein expression levels of PD-1 and PD-L1 in the lung (28). They demonstrated that silica exposure resulted in altered proportions and subtypes of T and B cells in immune organs, as well as the abnormalities of PD-1, PD-L1, and CTLA-4 expressions on these cells, leading to an imbalanced systemic immune homeostasis. Taken together, PD-1 and PD-L1 are abnormally expressed on specific cell types in the lung of the pulmonary fibrosis murine model, indicating that the PD-1/PD-L1 axis is vital in lung fibrosis occurrence and disease progression.

## 4 The profibrotic role of the PD-1/PD-L1 axis in IPF

The immune regulatory role of the PD-1/PD-L1 axis in cancer immunity has been determined. However, its role in IPF remains controversial. The profibrotic environment of IPF is shaped by proliferating cells, immune cells, stromal cells, growth factors, and extracellular matrix (ECM) proteins (37). It profoundly determines the onset of fibrosis and its ultimate destiny─ control or progression (37). According to currently published studies, the PD-1/PD-L1 pathway plays a double-edged sword effect in IPF. On the one hand, it can trigger lung fibrosis by interacting with multiple cell types and pathways. On the other hand, it could maintain immune homeostasis by interacting with the profibrotic component when fibrosis is initiated. Herein, we will first discuss its profibrotic role in IPF.

### 4.1 Th17 CD4^+^ T cells

There are numerous studies regarding the role of T helper 17 (Th17) cells in pulmonary fibrosis (38–52), with a consensus conclusion being made that they are profibrotic and detrimental in IPF (3). A previous study also observed elevated interleukin­17A (IL­17A), which is the typical cytokine of Th17 cells in the bronchoalveolar lavage (BALF) of IPF patients (53). Furthermore, animal studies illustrated that IL-17A deficiency could mitigate bleomycin and radiation-induced pulmonary fibrosis (54, 55). Sarcoidosis is one of the common idiopathic lung diseases. Braun and colleagues found significantly increased PD-1^+^ CD4^+^ T cells during sarcoidosis progression, with Th17 cells being the most predominant cell types expressing PD­1 (56). They revealed for the first time the relationship between the PD-1/PD-L1 pathway and sarcoidosis, providing insights for subsequent studies. Hence, in 2018, Celada et al. first investigated the role of PD-1^+^ CD4^+^ T cells in IPF and elucidated the intrinsic mechanisms (25). By conducting molecular, immunohistochemical, and FCM analyses of IPF patients and murine specimens, they identified that PD-1^+^ CD4^+^ T cells (mainly Th17 subsets) promote pulmonary fibrosis *via* signal transducer and activator of transcription 3 (STAT3)-mediated IL-17A and transforming growth factor–β (TGF-β) production. Interestingly, blockading the PD-1 pathway significantly decreased STAT3, IL-17A, and TGF-β expression levels on Th17 cells, subsequently reducing collagen I production from fibroblasts and attenuating lung fibrosis in a murine model. Consistent results were also verified in a later study (30).

Previous publications demonstrated that the upregulation of PD-1 on CD4^+^ T cells shaped an immunosuppressive environment in sarcoidosis patients and was correlated with disease aggravation in these individuals (56). Nevertheless, Celada et al. indicated that except for the known immune regulation role of PD-1^+^CD4^+^T cells, PD-1 upregulation on CD4^+^T cells also served a profibrotic role (25). It is well known that STAT3 could regulate PD-1 expression on T cells in various malignancies (57, 58). An increasing body of evidence recently suggests that PD-1 could also regulate STAT3 in pulmonary fibrosis and sarcoidosis (25, 30). Mechanically, STAT3 transcription activity could be negatively regulated by phosphatidylinositol 3­kinase (PI3K) (59). This pathway may be correlated with PD-1 manipulation of STAT3 expression in patients with sarcoidosis since a recent study showed that PD- 1 inhibits PI3K expression in CD4^+^ T cells in sarcoidosis (60). Therefore, the overexpressed PD-1 on Th17 cells might increase STAT3 transcription activity through indirect inhibition of PI3K (25). Besides, there is also a possibility that exosomes derived from Th17 cells could also regulate PD-1 expression (25).

**Figure 1:**
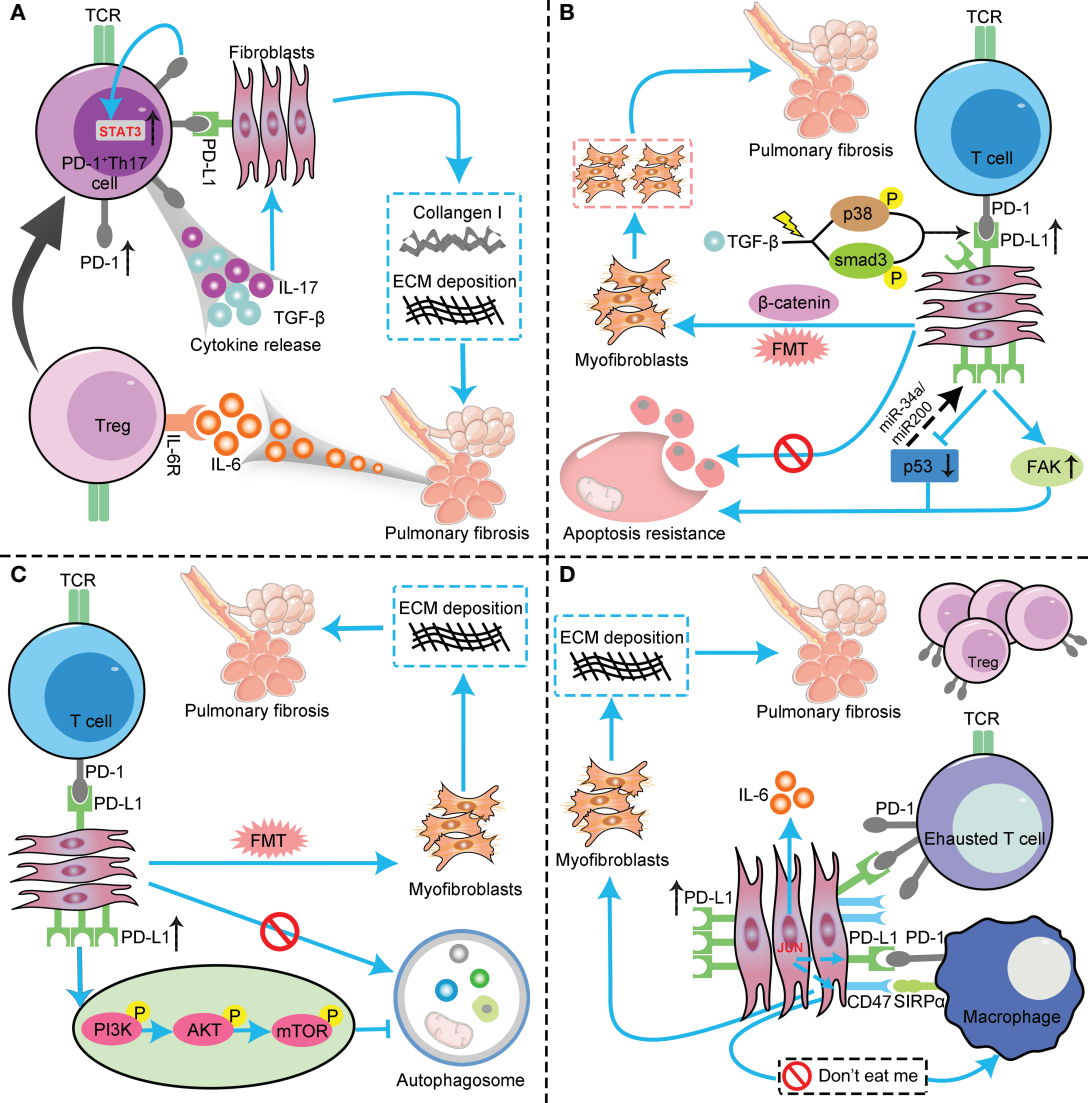
The profibrotic role of the PD-1/PD-L1 axis in IPF through interaction with multiple cell types and pathways. **(A)** PD-L1 up-regulation on Th17 T cells promotes pulmonary fibrosis through STAT3-mediated IL-17 and TGF-β production; **(B)** PD-L1 up-regulation on lung fibroblasts promotes pulmonary fibrosis *via* p53, FAK, Smad3, and β−catenin signaling pathways. On the one hand, PD-L1 up-regulation on lung fibroblasts may cause myofibroblasts to apoptosis-resistance and evasion phagocytosis *via* macrophages by inhibiting the p53 pathway and activating the FAK pathway, ultimately leading to excessive proliferation of myofibroblasts to trigger IPF. On the other hand, PD−L1 mediates lung fibroblast to myofibroblast transition (FMT) through Smad3 and β−catenin signaling pathways, thus promoting pulmonary fibrosis; **(C)** PD-L1 up-regulation on lung fibroblasts could induce myofibroblasts proliferation and ECM deposition through inhibiting autophagy, and eventually promotes pulmonary fibrosis; **(D)** PD-L1 up-regulation on lung fibroblasts promotes pulmonary fibrosis by inhibiting adaptive immunity. JUN upregulates the expression levels of PD-L1 and CD47 in fibroblasts and dormant macrophages. As a result, the above cells are converted into exhausted T cells and quiescent macrophages. In this context, myofibroblasts can evade immune clearance and resist macrophage-induced phagocytosis. In addition, JUN can also directly regulate IL-6 at the chromatin level, leading to inhibitory adaptive immune responses— primarily T cell exhaustion and upregulation of activated Tregs. PD-1, programmed cell death 1; PD-L1, programmed death-ligand 1; IPF, idiopathic pulmonary fibrosis; Th17, T helper 17; STAT3, signal transducer and activator of transcription 3; IL-17, interleukin-17; TGF-β, transforming growth factor–β; FAK, focal adhesion kinase; ECM, extracellular matrix; Tregs, regulatory T cells ; TCR, T cell receptor; FMT, fibroblast to myofibroblast transition; AKT, protein kinase B; PI3K, phosphoinositide 3-kinase; mTOR, mammalian target of rapamycin.

Both Th17 and regulatory T cells (Tregs) have been described as exhibiting the property of plasticity (61). Tregs upregulate TGF­β has been well documented (62). Tregs can reacquire characteristics of Th17 cells when exposed to IL-6 with or without IL-1β and IL-23 (61, 63). The increased IL6 production was observed in PD-1^+^Th17 cells from IPF patients (25). Besides, Th17 cells that secrete intracellular and membrane-bound TGF-β were also detected in IPF patients’ lung samples (25). Thus, it is reasonable that increased IL6 secretion caused Tregs to differentiate into Th17 subsets, thereby increasing IL17 and TGF-β expression levels and ultimately promoting pulmonary fibrosis (**Figure 1A**).

### 4.2 Fibroblasts/Myofibroblasts

The pathological process of IPF is intimately related to chronic lung injury and thereby causes a chronic inflammatory response (64). The cytokines and inflammatory mediators produced in this process could hasten the proliferation and differentiation of fibroblasts (64). As a result, fibroblasts are transformed into myofibroblasts, which are responsible for excessive proliferation, epithelial-mesenchymal transition (EMT), ECM deposition, and ultimately resulting in collagen overproduction in the lung (65). Recently, mounting evidence revealed that PD-L1 is upregulated on human and murine lung fibroblasts (17, 20, 21, 29). Most strikingly, these studies indicated that upregulated PD-L1 on lung fibroblasts entitled an invasive phenotype to fibroblasts, thereby promoting IPF progression, and anti-PD-L1 monoclonal antibody (anti-PD-L1 mAb) could reverse this effect. Thus, PD-L1 on lung fibroblasts contributes to pulmonary fibrosis occurrence and progression and may be a novel target for IPF treatment. However, the underlying mechanisms need to be well elucidated.

#### 4.2.1 p53 and FAK pathways

Recently, using RNA-seq analysis, Geng and colleagues revealed that PD-L1 was upregulated on invasive lung fibroblasts and was associated with the invasive phenotype of lung fibroblasts, is regulated by p53 and focal adhesion kinase (FAK) pathways, and drives lung fibrosis in a humanized pulmonary fibrosis murine model (21). *In vivo* and *in vitro* experiments have shown that the invasion ability of invasive lung fibroblasts and the severity of pulmonary fibrosis in mice could be attenuated by knocking out PD-L1 in fibroblasts and targeting PD-L1 using anti-PD-L1 mAb and FAK inhibitor.

The tumor suppressor protein p53 plays a crucial role in cell cycle arrest, apoptosis, senescence, and innate immunity (21, 66–70). Numerous studies have shown that the loss function of p53 is related to lung injury and IPF progression (21, 71–73). Besides, mounting evidence also suggests that the expression level of p53 is reduced in myofibroblasts compared to normal lung fibroblasts (74, 75). Emerging evidence indicated a negative regulatory loop between PD-L1 and p53 in invasive lung fibroblasts (21). We all know that p53 could regulate PD-L1 expression in malignancies *via* multiple members of microRNA families, such as miR-34a (76), miR-200 (77), and miRNA-320a (78). It suggests that p53 might contribute to PD-L1 regulation in lung fibroblasts. However, although molecular experiments have demonstrated that inhibition of PD-L1 could upregulate p53 expression level in IPF lung fibroblasts, the underlying mechanisms are still not well elucidated (21). A dual effect of p53 during normal wound healing has been acknowledged (78). Initially suppressed, it reemerged after healing and reached its peak after reepithelialization (79). On the contrary, myofibroblasts emerge in response to tissue injury and undergo apoptosis at wound closure (80). So, whether p53 determines the fate of myofibroblasts and whether gaining p53 function in myofibroblasts impacts fibrotic lung restoration? Recently, Qu and colleagues revealed that p53 functional restoration sensitizes lung myofibroblasts to apoptosis, promotes the clearance of apoptotic myofibroblasts by macrophages, and eventually results in lung fibrosis resolution in the pulmonary fibrosis murine model (75). Therefore, PD-L1 on lung myofibroblasts may cause myofibroblasts to apoptosis-resistance and evasion phagocytosis *via* macrophages by inhibiting the p53 pathway, ultimately leading to excessive proliferation of myofibroblasts to trigger IPF (**Figure 1B**).

FAK plays a crucial role in cell survival, proliferation, migration, and adhesion. Lung epithelial cell FAK signaling determines the ultimate destiny of lung epithelial cells and inhibits lung injury and fibrosis. The pharmaceutical intervention of FAK could dramatically inhibit the proliferation of lung myofibroblasts *in vitro* and attenuate the severity of lung fibrosis in IPF murine models *in vivo*. Interestingly, a recent study showed a positive correlation between PD-L1 and FAK mRNA expression levels in PD-L1-positive triple-negative breast cancer (TNBC) (81). Taken together, PD-L1 upregulation on lung fibroblasts aggravates IPF by inhibiting the p53 pathway to allow myofibroblasts to escape macrophage-induced apoptosis. Besides, it also aggravates fibrosis by augmenting the invasive phenotype of fibroblasts by regulating the FAK pathway (**Figure 1B**).

#### 4.2.2 Smad3 and β−catenin pathways

Recently, Guo and colleagues added new evidence for how PD-L1 on fibroblasts regulates the progression of pulmonary fibrosis (17). *In vitro* and *in vivo* studies demonstrated that PD-L1 is essential in the transition from fibroblast to myofibroblast, and PD-L1 acts through both Smad3-dependent and independent pathways to promote pulmonary fibrosis induced by TGF-β. Smad3 is a crucial transcription factor involved in TGF-β-induced IPF (82). Previous publications indicated that, except for the tumor cell surface, PD-L1 is also expressed in the nuclei of some malignancies (83, 84). Furthermore, treating TGF-β could upregulate PD-L1 expression levels in the nuclei of primary human lung fibroblasts. Ultimately, they identified that PD-L1 might act as a co-transactivator of TGF-β to enhance fibrotic marker gene expression and thus promote pulmonary fibrosis. A previous study elucidated that upregulation of PD-L1 could promote tumor growth and progression by activating the β-catenin pathway (85). Besides, aberrant activation of the GSK3β/β-catenin signaling pathway has been shown to correlate with IPF progression (86). Guo et al. demonstrated that β-catenin signaling was involved in the TGF-β-induced fibroblasts to myofibroblasts transition. Furthermore, they revealed that PD-L1 was involved in TGF-β-induced phosphorylation and inhibition of GSK3β at Serine 9, therefore inhibiting β-catenin degradation (17). Collectively, they identified that PD-L1 might also mediate TGF-β-induced fibroblasts to myofibroblasts transition through the β-catenin signaling (17). Therefore, PD−L1 upregulation on lung fibroblasts may promote IPF progression *via* Smad3 and β−catenin signaling pathways. Mechanically speaking, the activation of TGF-β could recruit and phosphorylate Smad3 by activating the receptor kinases. Activated Smad3 then translocates into the nucleus, which binds with other co-factors to activate fibrotic-related gene transcription. Besides, the downstream Smad3 and p38 pathways of TGF-β could upregulate the PD-L1 expression level on the lung fibroblasts. As a result, the upregulated PD-L1 binds to Smad3 and enhances its transcription activation of fibrotic-related genes. Furthermore, PD-L1 also facilitates GSK3β phosphorylation at Ser9, thus inhibiting GSK3β-dependent degradation of β-catenin. Increased β-catenin may also contribute to TGF-β-induced fibrotic-related gene expression through binding with the T cell factor (TCF) transcription factor (**Figure 1B**).

#### 4.2.3 Autophagy

Autophagy is a highly conserved and pivotal catabolic process in eukaryotic cells (87, 88). It maintains the homeostasis of the intracellular environment by forming autophagosome vesicles, engulfing dysfunctional cytoplasm and organelles, and forming autolysosomes to degrade the contents of vesicles (89, 90). Autophagy disorder involves various diseases, also including IPF (91–96). Accumulating evidence revealed that autophagy activity was impaired in IPF, and inhibition of autophagy could induce myofibroblast proliferation and ECM deposition, thus leading to fibrosis (97, 98). On the contrary, pharmaceutical administration of autophagy activators could attenuate fibrotic severity (99). Interestingly, a recent study indicated that anti-PD-L1 mAb significantly inhibited the invasive ability and ECM deposition of TGF-β1-induced lung fibroblasts by downregulating the PI3K/Akt/mTOR signaling pathway to induce autophagy (29). It is well elucidated that PI3K/Akt signal pathway plays a pivotal role in regulating cell growth, proliferation, motility, metabolism, and survival (1). Most importantly, recent studies have identified that PI3K/Akt activation significantly correlated with the expression of the fibrotic-related gene alpha-smooth muscle actin (α-SMA) (100). Further, it is also suggested that the interaction between PI3K/Akt and TGF-β involves pulmonary fibrosis formation (101). The mammalian target of rapamycin (mTOR) is downstream of the PI3K/Akt signal pathway. Activation of the PI3K/Akt signaling pathway could activate mTOR, which inhibits autophagy in myofibroblasts and ultimately promotes the formation of pulmonary fibrosis (102) (**Figure 1C**).

Recently, several studies have revealed subtle crosstalk between PD-L1 and autophagy in cancer cells (103, 104). In mouse melanoma and human ovarian cancer, tumor cell-intrinsic PD-L1 upregulates mTOR complex 1 signaling to inhibit autophagy and sensitizes tumor cells to clinically available autophagy inhibitors (103). Meanwhile, another study demonstrated that autophagy regulates PD-L1 expression in gastric cancer through the p62/SQSTM1-NF-κB pathway (104). *In vitro* and *in vivo* studies indicated that inhibition of autophagy upregulated the expression of PD-L1 in gastric cancer cells. Hence, there might be an interaction between PDL1 expression on myofibroblasts and autophagy in IPF, and they could be novel biomarkers and therapeutic targets in IPF diagnosis and treatment. Besides, more relevant studies are warranted to make this clear.

#### 4.2.4 Inhibition of adaptive immunity

Adaptive immunity has been shown to orchestrate existing fibrotic responses, and various subsets of T cells are enriched in fibrotic lungs (20). In a recent review, Shenderov and colleagues demonstrated that immune dysregulation served as a driver of IPF, and several cell types were involved in this process (3). They indicated that M2 macrophages, Th17 cells, CD8^+^T cells, and possibly Tregs promote fibrosis, while Th1 and tissue-resident memory (TRM) CD4^+^ T cells appear to be protective (3). Besides, there is growing evidence that increased levels of activated Tregs infiltration are associated with disease progression in pulmonary fibrosis (105–107). As an essential immune checkpoint molecule, the immunosuppressive role of PD-L1 in cancer immunity is elucidated. In a recent publication, Cui et al. revealed that fibroblasts PD-L1 also elicit adaptive immunity dysfunction in the transcription factor JUN inducted pulmonary fibrosis murine model (20). By employing a single-cell protein screening approach in human and murine fibrotic lungs, JUN expression in fibroblasts increased IL-6 expression and secretion, which plays pivotal roles in adaptive and innate immunity. Besides, JUN expression in fibroblasts also upregulated the expression levels of PD-L1 and CD47, suggesting that these two immune regulatory pathways are dysregulated in IPF. Surprisingly, they identified that blockade of PD-L1, CD47, and IL-6 significantly alleviated the severity of pulmonary fibrosis not only in bleomycin-induced pulmonary fibrosis mice model but in IL-6 knockout and humanized NSG mice models. CD47 is a crucial molecule that mediates malignant cells or impaired cells to resist phagocytosis (108, 109). JUN expression in lung myofibroblasts directly controls the promoters and enhancers of CD47 and PD-L1. The direct consequence is increased expression of these immune-checkpoint proteins in fibroblasts and dormant macrophages. Therefore, these immunomodulatory pathways are hyperactivated. As a result, in the fibrotic environment, T cells and macrophages become exhausted and quiescent. In this context, myofibroblasts could evade immune clearance and resist macrophage-induced phagocytosis. In addition, JUN could also directly regulate IL-6 at the chromatin level, which results in the inhibitory adaptive immune response─ chiefly T-cell exhaustion and upregulation of activated Tregs. Ultimately, pulmonary fibrosis initiates and continues to worsen in this vicious circle (**Figure 1D**).

## 5 The immune regulatory role of the PD-1/PD-L1 axis in IPF

As mentioned in the previous section, current evidence indicates that the PD-1/PD-L1 pathway has dual effects in the IPF─ profibrotic and immune regulatory roles. We have discussed the profibrotic role of the PD-1/PD-L1 pathway in IPF initiating and disease progression. Here, we will review its immune regulatory role in IPF.

### 5.1 CD28^null^ T cells

Although the exact role of the immune system in the development and progression of IPF is currently under debate, existing evidence suggests that this disease is associated with the hyperactivation of immune-related pathways (26, 110) and aberrant infiltration of some subsets of immune cells (111). Clinical investigations have demonstrated abnormal infiltration of T cells in the lung tissue of patients with IPF (22, 112). Currently, the phenotype of T cells in IPF is not well characterized (26). It has been reported that one or more co-stimulatory molecules, such as CD28 and ICOS receptors, are absent on the surface of T cells in the peripheral blood of IPF patients (113, 114). Furthermore, several studies have shown that CD28^null^ T cell abundance in T cells is associated with the bleak prognosis of these individuals (113, 114).

CD28^null^ T cells are antigen-experienced memory T cells present in the pathological process of various diseases (26). These cells have been observed to share similar phenotypes with CD8^+^ cytotoxic T cells (115, 116). In a recent study, Habiel and colleagues identified that the populations of CD8^+^ CD28^null^ T cells in explanted lung cellular suspensions from IPF patients were significantly elevated than normal donor lungs (26). *In vivo* study indicated that IPF CD28^null^ T cells may promote dexamethasone-resistant lung fibrosis. Besides, they observed that intravenous administration of CD28^null^-enriched T cells purified from IPF lung explants could induce severe lung remodeling by targeting alveolar type II epithelial cells or by modulating surfactant protein C production by these cells in humanized NSG mice model. Most importantly, FCM and transcriptional analysis detected higher levels of PD-1 and CTLA-4 on CD28^null^ cytotoxic T cells relative to CD28^+^ cytotoxic T cells. Furthermore, it showed that PD-L1 was overexpressed on structural cells in IPF lungs compared to normal lung samples. Interestingly, anti-CTLA-4 or anti-PD-1 mAb intervention significantly exuberated the severity of pulmonary fibrosis in humanized NSG mice. Therefore, IPF CD28^null^ T cells may serve as a profibrotic component in lung fibrosis, but the immune checkpoint molecules CTLA-4 and PD-1 appear to limit this effect.

### 5.2 Mesenchymal stem cells

MSCs are multipotent stromal cells in multiple human tissues and are characterized by differentiating into mesodermal lineage cells (117, 118). Human MSCs have immunomodulation capacities and have been proven to regulate the activity and function of major immune cell populations, including T cells (22, 119). As such, human MSCs have brought light to cell therapy and tissue regeneration in many diseases, also including pulmonary fibrosis (22, 120). Recently, Ni et al. observed that lymphocytes, especially CD8^+^ T cells, were overactivated at the early stage of bleomycin administration in a humanized NSG pulmonary fibrosis mice model (22). At the late stage, myofibroblasts were activated and accompanied by ECM deposition and lung reconstruction, suggesting the occurrence of IPF. Most strikingly, human MSCs intervention could attenuate the severity of pulmonary fibrosis and improve lung function *via* inhibiting bleomycin-induced T cell infiltration and pro-inflammatory cytokine production in the humanized mice model. Ultimately, they revealed that the PD-1/PD-L1 pathway mediated the alleviation of pulmonary fibrosis by human MSCs.

Human MSCs only play a constitutively immunomodulatory role with proper licensing from the inflammatory environment, especially *in vivo* (121). In the humanized pulmonary fibrosis mice model, the pro-inflammatory microenvironment during pulmonary fibrosis, characterized by high inflammatory cytokines production and high infiltration of CD8^+^ T cells, could maintain the immune regulatory ability of human MSCs (122). In response, human MSCs might express immunomodulatory factors to restrict the profibrotic effect to the lung (22). As one of the most crucial inhibitory immune checkpoints expressed on T cells, once PD-1 is engaged with its ligand PD-L1, it can inhibit the PI3K/Akt pathway and the phosphorylation of ZAP70 and PKCθ and subsequently suppress T cell activation by inhibiting TCR signaling (123). As mentioned before, PD-L1 is also expressed on MSCs. Ni and colleagues demonstrated that PD-1 is significantly overexpressed on T cells in the humanized pulmonary fibrosis mice model, while PD-L1 is highly expressed on activated MSCs (22). Besides, they observed that the interaction of PD-1 and PD-L1 was involved in the immunosuppression of IFN-γ-licensed human MSCs on T cell activation *in vitro*. Hence, PD-L1 upregulated on human MSCs could engage with PD-1 on the CD8^+^T cells in the profibrotic environment of the lung, subsequently delivering an inhibitory signal to the immune system to limit CD8^+^T cells’ hyperactivation and pro-inflammatory cytokines secretion. However, there are still some issues that deserve our attention. First, although *in vitro* study indicated that human MSCs have an anti-fibrotic role in IPF *via* the immunomodulatory role of the PD-1/PD-L1 axis, whether the PD-1/PD-L1 axis is involved in human MSC immunoregulation *in vivo* remains unclear. Second, the above study showed that human MSCs only bring therapeutic benefits at the early stage of pulmonary fibrosis. Third, the profibrotic environment is complicated and shaped by multiple cell types and factors. The author only highlighted the role of PD-L1 on human MSCs. However, the fact is that PD-L1 is also expressed on other cells in the lung of IPF patients and murine models (such as myofibroblasts). Taken together, the PD-1/PD-L1 axis participates in ameliorating pulmonary fibrosis by human MSCs in the humanized pulmonary fibrosis mice model. Considering the complexity of the fibrotic environment in IPF and the administration time of human MSCs, relevant studies should be performed to address the above problems.

## 6 Is it rational to target the PD-1/PD-L1 axis for IPF treatment?

Studies in individuals with IPF and mouse models suggest that the PD-1/PD-L1 axis has a more predominant profibrotic role than its immunomodulatory role in IPF. Thus, numerous preclinical studies were conducted to investigate the feasibility of treating IPF by targeting the PD-1/PD-L1 axis in mice models (20, 21, 25, 26, 28, 29). The majority of studies identified that blockade of the PD-1/PD-L1 axis using PD-L1 mAb could attenuate the severity of pulmonary fibrosis in mice models. Interestingly, as we mentioned before, IPF and lung cancer share common bio-molecular characteristics, and the PD-1/PD-L1 axis is one of the common pathways between them. Recently, one study investigated the therapeutic role of pirfenidone combined with PD-L1 blockade in the pulmonary fibrosis-lung cancer mice model (124). They observed that this combination treatment significantly facilitated the infiltration of immune cells, delayed tumor growth, and improved the prognosis of the mice. Most excitingly, combination therapy attenuated the pulmonary fibrosis of the mice. Therefore, combining anti-fibrotic agents with ICIs may bring potential benefits for IPF. Nevertheless, not all combination therapies are beneficial for relieving pulmonary fibrosis, and sometimes it could be hazardous. For instance, human MSCs contribute to immunomodulatory and are employed in IPF treatment in many preclinical studies (125). However, the combination of human MSCs and PD-1/PD-L1 inhibitors should be avoided since animal studies implicated that the administration of PD-1/PD-L1 inhibitors could reverse the anti-fibrotic role of human MSCs (22).

Although current studies indicated that the PD-1/PD-L1 axis plays a crucial role in promoting IPF, this does not imply that ICIs targeting the PD-1/PD-L1 pathway could become a new strategy for treating IPF in clinical practice. The progression of fibrosis is determined by a dynamic balance between anti-fibrotic and profibrotic mediators within a microenvironment composed of diverse cellular subtypes (20). According to current studies, PD-L1 downregulation on fibroblasts could relieve pulmonary fibrosis through multiple pathways. However, the subsequent immune response due to T cell hyperactivation might break this balance. Up to now, the role of immune cells and immune cell activation in IPF remains controversial (26). However, increased CD8^+^ T cells in the lung and the airway of IPF patients were found to correlate with severe pulmonary function damage (126, 127). Most importantly, current clinical trials revealed that anti-PD-1/PD-L1 agents increase the risk for severe and potentially life-threatening adverse effects in cancer patients (128). Among them, the incidence of immune checkpoint inhibitor-associated pneumonitis is staggering at 3% to 10% in cancer patients (128). It is worth noting that pre-existing pulmonary fibrosis is a commonly recognized risk factor for immune checkpoint inhibitor-associated pneumonitis (129). Therefore, targeting the PD-1/PD-L1 axis to mitigate pulmonary fibrosis might be a novel insight into IPF treatment. However, there is still a long way to go before finding an optimal balance between immunity activation and PD-L1 degradation.

## 7 Conclusion and prospects

Despite the advances in IPF pathogenic mechanism and treatment that have been achieved in recent decades, it is still a lethal disease and needs novel therapeutic strategies to be developed. In recent years, with the discovery of bio-molecular similarities between IPF and lung cancer and the role of the PD-1/PD-L1 axis in cancer immunity, more and more studies have begun to focus on the role of the PD-1/PD-L1 axis in IPF. *In vitro* and *in vivo* studies demonstrated that the PD-1/PD-L1 axis plays a more predominant profibrotic role than its immune regulatory role in IPF by interacting with multiple cell types and pathways. Most murine studies indicated that blockade of the PD-1/PD-L1 axis could attenuate pulmonary fibrosis and improve the prognosis of pulmonary fibrosis mice. Although the pathogenesis of IPF remains largely unknown, the current findings imply that the PD-1/PD-L1 pathway might be a candidate therapeutic target for IPF treatment in the future.

Although we comprehensively summarized the role of the PD-1/PD-L1 axis in IPF for the first time and provided potential insights into this field, the following issues need to be well investigated in future studies. First, despite the majority of published studies illustrating that the PD-1/PD-L1 axis serves a profibrotic role in IPF and pharmaceutical intervention of this pathway could alleviate pulmonary fibrosis, all results were obtained based on murine studies, and the evidence from clinical studies remains lacking. Besides, it is growing recognized that cytokine IL-6 plays a crucial role in pulmonary fibrosis (130). Meanwhile, the preclinical study demonstrated that the blockade of IL-6, CD47, and PD-L1 together could ameliorate pulmonary fibrosis by increasing phagocytosis of profibrotic fibroblasts and by eliminating suppressive effects on adaptive immunity (20). As we know, a clinically tested blocking antibody against IL-6 is available, and the Food and Drug Administration (FDA) approved it for rheumatoid arthritis and acute cytokine release syndrome treatment (130, 131). Many pharmaceutical companies also developed reagents to target both CD47 and PD-L1 immune checkpoint molecules, and a growing number of studies are also conducted to evaluate the therapeutical potential of antibodies that target CD47 and PD-L1 in various malignancies (132–137). How about the effect of IL-6 antibody combination with CD47 and PD-L1 ICIs in patients with IPF or lung cancer combined with IPF? Clinical studies could be designed to answer this question. Furthermore, both PD-1 and PD-L1 exist in two forms: membrane bound (mPD-L1) and soluble (sPD-L1) forms (138, 139). Recent studies reported that sPD-1 and sPD-L1 could be detected in the plasma or serum of patients with lung cancer (138, 140, 141). Meanwhile, sPD-L1 was associated with the prognosis and treatment response in lung cancer (138, 140, 141). To our regret, there were only two small sample size studies reported the abnormal elevation of sPD-L1 in the serum of IPF patients. Hence, large scale prospective studies could be designed to detect the expression levels of sPD-L1 and sPD-1 in IPF patients and explore their prognostic significance in these patients. Second, IPF is a highly complex disease, and multiple factors participate in its occurrence and progression. For instance, it is well documented that lung macrophages and its subpopulations play pivotal roles in pulmonary fibrosis pathogenesis (142–145). Recently, Hartley and colleagues investigated PD-L1 signaling in macrophages and the effects of PD-L1 antibody treatment on tumor-associated macrophages (TAM) responses (146). They elucidated that PD-L1 delivers a constitutive negative signal to macrophages, resulting in an immune-suppressive cell phenotype. Treatment with PD-L1 antibodies reverses this phenotype and triggers macrophage-mediated antitumor activity. However, there was only one study directly reported the role of the crosstalk between the PD-1/PD-L1 pathway and lung macrophages in the pathogenesis of pulmonary fibrosis in our review (20). Hence, more relevant studies could be designed to provide more insights into this field. Besides, the contributions of diverse cell populations in the lung to pulmonary fibrosis pathogenesis are poorly understood. Recently, as an innovative technology, single-cell RNA sequencing (scRNA-*seq*) has been adopted to investigate the transcriptome heterogeneity of different cell types in many diseases (147), also including IPF (20, 145, 148–157). Taking advantage of genomics technology, especially the advances of scRNA-*seq* in recent decades, relevant studies could be designed to confirm the role of the PD-1/PD-L1 axis in IPF, identify the involved cell populations, and clarify the underlying regulatory mechanisms. Third, we identified that the PD-1/PD-L1 axis plays a more predominant profibrotic role than its immune regulatory role in IPF. It appears that lung fibroblast/myofibroblast PD-L1 upregulation is at the core of this process. Therefore, is there a role of PD-L1 degradation in treating IPF? Until now, genetic knockout strategies, including RNA interference (RNAi), antisense oligonucleotides, and CRISPR/Cas9 are the most explored methods to decrease cellular protein levels (158). Recently, chemical knockout strategies have emerged as promising approaches because of their improved efficacy and reduced side effects (158). Among them, as a novel therapeutic strategy to target traditionally “undruggable” proteins, proteolysis targeting chimeras (PROTACs) have been widely used to deplete a protein of interest (35, 159–161). Recently, Cotton and colleagues designed an antibody-based PROTACs (AbTACs) that could induce the lysosomal degradation of PD-L1 in multiple cancer cell lines by recruiting the membrane-bound E3 ligase RNF43 (159). Nevertheless, targeted degradation of membrane PD-L1 is still challenging for PROTACs. To overcome this bottleneck, Wang et al. recently developed a novel strategy that could achieve precise degradation of the membrane protein PD-L1 in cancer cells by using enzyme-instructed self-assembly (EISA) and surface-induced self-assembly (158). Inspired by the successful applications of the targeted protein degradation technology in this field, is it feasible to use these strategies to selectively degrade PD-L1 in lung fibroblasts/myofibroblasts for IPF treatment? We think relevant preclinical studies could be designed to verify its feasibility. Furthermore, with the rapid development of material science today, especially the insightful understanding of nanomedicine, many nano-drugs and nanotechnologies are developed for disease diagnosis and treatment (162–165). In this context, many approaches and designs have been developed to target the PD-1/PD-L1 axis in tumors to improve the efficacy of immunotherapy (166). Many active agents such as antibodies, peptides, siRNAs, miRNAs, and small molecules have been routinely incorporated into various nanosystems for therapeutic, targeting, or diagnostic purposes (166, 167). Given the crucial role of the PD-1/PD-L1 pathway in IPF, will it be a feasible strategy to apply the above nanomedicines or methods for treating IPF? It will be challenging and needs a long way to go.

## Author contributions

YY, WZ, and AJ conceived and designed the manuscript, provided guidance, and edited the manuscript. AJ wrote the manuscript. All authors contributed to the article and approved the submitted version.

## Funding

This work was financially supported by the Key Research and development program of Shaanxi Province (No. 2020GXLH-Y020, for YY), the National Natural Science Foundation of China (No. 82141126), and The Innovation Capability Program of Shaanxi (No. 2021TD-44).

## Conflict of interest

The authors declare that the research was conducted in the absence of any commercial or financial relationships that could be construed as a potential conflict of interest.

## Publisher’s note

All claims expressed in this article are solely those of the authors and do not necessarily represent those of their affiliated organizations, or those of the publisher, the editors and the reviewers. Any product that may be evaluated in this article, or claim that may be made by its manufacturer, is not guaranteed or endorsed by the publisher.

## References

[1] Wang J Hu K Cai X Yang B He Q Wang J. Targeting PI3K/AKT signaling for treatment of idiopathic pulmonary fibrosis. Acta Pharm Sin B (2022) 12(1):18–32. doi: 10.1016/j.apsb.2021.07.023 35127370 PMC8799876

[2] Ahmadvand N Carraro G Jones MR Shalashova I Noori A Wilhelm J. Cell-surface programmed death ligand-1 expression identifies a Sub-population of distal epithelial cells enriched in idiopathic pulmonary fibrosis. Cells (2022) 11(10):1593. doi: 10.3390/cells11101593 35626630 PMC9139571

[3] Shenderov K Collins SL Powell JD Horton MR. Immune dysregulation as a driver of idiopathic pulmonary fibrosis. J Clin Invest (2021) 131(2):e143226. doi: 10.1172/JCI143226 33463535 PMC7810481

[4] King TE Jr. Bradford WZ Castro-Bernardini S Fagan EA Glaspole I Glassberg MK. A phase 3 trial of pirfenidone in patients with idiopathic pulmonary fibrosis. N Engl J Med (2014) 370(22):2083–92. doi: 10.1056/NEJMoa1402582 24836312

[5] Richeldi L Du Bois RM Raghu G Azuma A Brown KK Costabel U. Efficacy and safety of nintedanib in idiopathic pulmonary fibrosis. N Engl J Med (2014) 370(22):2071–82. doi: 10.1056/NEJMoa1402584 24836310

[6] Raghu G Rochwerg B Zhang Y Garcia CA Azuma A Behr J. An official ATS/ERS/JRS/ALAT clinical practice guideline: Treatment of idiopathic pulmonary fibrosis. an update of the 2011 clinical practice guideline. Am J Respir Crit Care Med (2015) 192(2):e3–19. doi: 10.1164/rccm.201506-1063ST 26177183

[7] Tzouvelekis A Gomatou G Bouros E Trigidou R Tzilas V Bouros D. Common pathogenic mechanisms between idiopathic pulmonary fibrosis and lung cancer. Chest (2019) 156(2):383–91. doi: 10.1016/j.chest.2019.04.114 31125557

[8] Kim HC Lee S Song. JW. Impact of idiopathic pulmonary fibrosis on clinical outcomes of lung cancer patients. Sci Rep (2021) 11(1):8312. doi: 10.1038/s41598-021-87747-1 33859288 PMC8050293

[9] Tomassetti S Gurioli C Ryu JH Decker PA Ravaglia C Tantalocco P. The impact of lung cancer on survival of idiopathic pulmonary fibrosis. Chest (2015) 147(1):157–64. doi: 10.1378/chest.14-0359 25166895

[10] Pardoll DM. The blockade of immune checkpoints in cancer immunotherapy. Nat Rev Cancer (2012) 12(4):252–64. doi: 10.1038/nrc3239 PMC4856023 22437870

[11] Yi M Niu M Xu L Luo S Wu K. Regulation of PD-L1 expression in the tumor microenvironment. J Hematol Oncol (2021) 14(1):10. doi: 10.1186/s13045-020-01027-5 33413496 PMC7792099

[12] Qingyang L Wang D Sun K Wang L Zhang Y. Resistance mechanisms of anti-PD1/PDL1 therapy in solid tumors. Front Cell Dev Biol (2020) 8:672. doi: 10.3389/fcell.2020.00672 32793604 PMC7385189

[13] O'malley G Treacy O Lynch K Naicker SD Leonard NA Lohan P. Stromal cell PD-L1 inhibits CD8(+) T-cell antitumor immune responses and promotes colon cancer. Cancer Immunol Res (2018) 6(11):1426–41. doi: 10.1158/2326-6066.Cir-17-0443 30228206

[14] Beswick EJ Grim C Singh A Aguirre JE Tafoya M Qiu S. Expression of programmed death-ligand 1 by human colonic CD90(+) stromal cells differs between ulcerative colitis and crohn's disease and determines their capacity to suppress Th1 cells. Front Immunol (2018) 9:1125. doi: 10.3389/fimmu.2018.01125 29910803 PMC5992387

[15] Dezutter-Dambuyant C Durand I Alberti L Bendriss-Vermare N Valladeau-Guilemond J Duc A. A novel regulation of PD-1 ligands on mesenchymal stromal cells through MMP-mediated proteolytic cleavage. Oncoimmunology (2016) 5(3):e1091146. doi: 10.1080/2162402x.2015.1091146 27141350 PMC4839348

[16] Beswick EJ Johnson JR Saada JI Humen M House J Dann S. TLR4 activation enhances the PD-L1-mediated tolerogenic capacity of colonic CD90+ stromal cells. . J Immunol (2014) 193(5):2218–29. doi: 10.4049/jimmunol.1203441 PMC4142442 25070848

[17] Guo X Sunil C Adeyanju O Parker A Huang S Ikebe M. PD-L1 mediates lung fibroblast to myofibroblast transition through Smad3 and β-catenin signaling pathways. Sci Rep (2022) 12(1):3053. doi: 10.1038/s41598-022-07044-3 35197539 PMC8866514

[18] Wang B Bai W Ma H Li F. Regulatory effect of PD1/PD-ligand 1 (PD-L1) on treg cells in patients with idiopathic pulmonary fibrosis. Med Sci Monit (2021) 27:e927577. doi: 10.12659/msm.927577 33386384 PMC7786833

[19] Kronborg-White S Madsen LB Bendstrup E Poletti V. PD-L1 expression in patients with idiopathic pulmonary fibrosis. J Clin Med (2021) 10(23):5562. doi: 10.3390/jcm10235562 34884264 PMC8658518

[20] Cui L Chen SY Lerbs T Lee JW Domizi P Gordon S. Activation of JUN in fibroblasts promotes pro-fibrotic programme and modulates protective immunity. Nat Commun (2020) 11(1):2795. doi: 10.1038/s41467-020-16466-4 32493933 PMC7270081

[21] Geng Y Liu X Liang J Habiel DM Kulur V Coelho AL. PD-L1 on invasive fibroblasts drives fibrosis in a humanized model of idiopathic pulmonary fibrosis. JCI Insight (2019) 4(6):e125326. doi: 10.1172/jci.insight.125326 30763282 PMC6482997

[22] Ni K Liu M Zheng J Wen L Chen Q Xiang Z. PD-1/PD-L1 pathway mediates the alleviation of pulmonary fibrosis by human mesenchymal stem cells in humanized mice. Am J Respir Cell Mol Biol (2018) 58(6):684–95. doi: 10.1165/rcmb.2017-0326OC 29220578

[23] Jovanovic D Milenkovic MR Kotur Stevuljevic J Markovic J Ceriman V Kontic M. Membrane PD-L1 expression and soluble PD-L1 plasma levels in idiopathic pulmonary fibrosis-a pilot study. J Thorac Dis (2018) 10(12):6660–9. doi: 10.21037/jtd.2018.11.16 PMC6344764 30746211

[24] Roksandic Milenkovic M Klisic A Ceriman V Kotur Stevuljevic J Savic Vujovic K Mirkov D. Oxidative stress and inflammation parameters-novel biomarkers for idiopathic pulmonary fibrosis. Eur Rev Med Pharmacol Sci (2022) 26(3):927–34. doi: 10.26355/eurrev_202202_28002 35179759

[25] Celada LJ Kropski JA Herazo-Maya JD Luo W Creecy A Abad AT. PD-1 up-regulation on CD4(+) T cells promotes pulmonary fibrosis through STAT3-mediated IL-17A and TGF-β1 production. Sci Transl Med (2018) 10(460):eaar8356. doi: 10.1126/scitranslmed.aar8356 30257954 PMC6263177

[26] Habiel DM Espindola MS Kitson C Azzara AV Coelho AL Stripp B. Characterization of CD28(null) T cells in idiopathic pulmonary fibrosis. Mucosal Immunol (2019) 12(1):212–22. doi: 10.1038/s41385-018-0082-8 PMC6301115 30315241

[27] Geng Y Su S Cao L Yang T Ouyang W Liu L. Effect of PD-1 inhibitor combined with X-ray irradiation on the inflammatory microenvironment and lung tissue injury in mice. J Inflammation Res (2022) 15:545–56. doi: 10.2147/jir.S350112 PMC8803086 35115804

[28] Zhao Y Hao C Li M Qu Y Guo Y Deng X. PD-1/PD-L1 inhibitor ameliorates silica-induced pulmonary fibrosis by maintaining systemic immune homeostasis. BioMed Pharmacother (2022) 148:112768. doi: 10.1016/j.biopha.2022.112768 35247717

[29] Lu Y. W Liu Y Chen W Zhang J Zeng Z Huang H. Anti-PD-L1 antibody alleviates pulmonary fibrosis by inducing autophagy *via* inhibition of the PI3K/Akt/mTOR pathway. Int Immunopharmacol (2022) 104:108504. doi: 10.1016/j.intimp.2021.108504 35026657

[30] Wang D Gong L Li Z Chen H Xu M Rong R. Antifibrotic effect of gancao ganjiang decoction is mediated by PD-1 / TGF-β1 / IL-17A pathway in bleomycin-induced idiopathic pulmonary fibrosis. J Ethnopharmacol (2021) 281:114522. doi: 10.1016/j.jep.2021.114522 34391863

[31] Li S Shi J Tang H. Animal models of drug-induced pulmonary fibrosis: an overview of molecular mechanisms and characteristics. Cell Biol Toxicol (2021) 38(5):699–723. doi: 10.1007/s10565-021-09676-z 34741237

[32] Rangarajan S Bone NB Zmijewska AA Jiang S Park DW Bernard K. Metformin reverses established lung fibrosis in a bleomycin model. Nat Med (2018) 24(8):1121–7. doi: 10.1038/s41591-018-0087-6 PMC6081262 29967351

[33] Walsh NC Kenney LL Jangalwe S Aryee KE Greiner DL Brehm MA. Humanized mouse models of clinical disease. Annu Rev Pathol (2017) 12:187–215. doi: 10.1146/annurev-pathol-052016-100332 27959627 PMC5280554

[34] Yan J Zheng X You W He W Xu GK. A bionic-homodimerization strategy for optimizing modulators of protein-protein interactions: From statistical mechanics theory to potential clinical translation. Adv Sci (Weinh) (2022) 9(11):e2105179. doi: 10.1002/advs.202105179 35166067 PMC9008432

[35] Zheng X Yan J You W Li F Diao J He W. *De novo* nano-erythrocyte structurally braced by biomimetic Au(I)-peptide skeleton for MDM2/MDMX predation toward augmented pulmonary adenocarcinoma immunotherapy. Small (2021) 17(20):e2100394. doi: 10.1002/smll.202100394 33870652

[36] He W Zhang Z Yang W Zheng X You W Yao Y. Turing Milk into pro-apoptotic oral nanotherapeutic: *De novo* bionic chiral-peptide supramolecule for cancer targeted and immunological therapy. Theranostics (2022) 12(5):2322–34. doi: 10.7150/thno.70568 PMC8899570 35265212

[37] Lettieri S Oggionni T Lancia A Bortolotto C Stella GM. Immune stroma in lung cancer and idiopathic pulmonary fibrosis: A common biologic landscape? Int J Mol Sci (2021) 22(6):2882. doi: 10.3390/ijms22062882 33809111 PMC8000622

[38] Jia Q Li Q Wang Y Zhao J Jiang Q Wang H. Lung microbiome and transcriptome reveal mechanisms underlying PM(2.5) induced pulmonary fibrosis. Sci Total Environ (2022) 831:154974. doi: 10.1016/j.scitotenv.2022.154974 35378184

[39] Yang D Chen X Wang J Lou Q Lou Y Li L. Dysregulated lung commensal bacteria drive interleukin-17B production to promote pulmonary fibrosis through their outer membrane vesicles. Immunity (2019) 50(3):692–706.e7. doi: 10.1016/j.immuni.2019.02.001 30824326

[40] Martinu T Mcmanigle WC Kelly FL Nelson ME Sun J Zhang HL. IL-17A contributes to lung fibrosis in a model of chronic pulmonary graft-versus-host disease. Transplantation (2019) 103(11):2264–74. doi: 10.1097/tp.0000000000002837 PMC7550218 31658231

[41] Russo RC Savino B Mirolo M Buracchi C Germano G Anselmo A. The atypical chemokine receptor ACKR2 drives pulmonary fibrosis by tuning influx of CCR2(+) and CCR5(+) IFNγ-producing γδT cells in mice. Am J Physiol Lung Cell Mol Physiol (2018) 314(6):L1010–l25. doi: 10.1152/ajplung.00233.2017 29469612

[42] Chakraborty K Chatterjee S Bhattacharyya A. Impact of treg on other T cell subsets in progression of fibrosis in experimental lung fibrosis. Tissue Cell (2018) 53:87–92. doi: 10.1016/j.tice.2018.06.003 30060832

[43] Lei L Zhao C Qin F He ZY Wang X Zhong XN. Th17 cells and IL-17 promote the skin and lung inflammation and fibrosis process in a bleomycin-induced murine model of systemic sclerosis. Clin Exp Rheumatol (2016) 34 Suppl 100(5):14–22. 26750756

[44] Xiong S Guo R Yang Z Xu L Du L Li R. Treg depletion attenuates irradiation-induced pulmonary fibrosis by reducing fibrocyte accumulation, inducing Th17 response, and shifting IFN-γ, IL-12/IL-4, IL-5 balance. Immunobiology (2015) 220(11):1284–91. doi: 10.1016/j.imbio.2015.07.001 26224246

[45] Oh K Seo MW Kim YW Lee DS. Osteopontin potentiates pulmonary inflammation and fibrosis by modulating IL-17/IFN-γ-secreting T-cell ratios in bleomycin-treated mice. Immune Netw (2015) 15(3):142–9. doi: 10.4110/in.2015.15.3.142 PMC4486777 26140046

[46] Chen Y Li C Weng D Song L Tang W Dai W. Neutralization of interleukin-17A delays progression of silica-induced lung inflammation and fibrosis in C57BL/6 mice. Toxicol Appl Pharmacol (2014) 275(1):62–72. doi: 10.1016/j.taap.2013.11.012 24291675

[47] Song L Weng D Liu F Chen Y Li C Dong L. Tregs promote the differentiation of Th17 cells in silica-induced lung fibrosis in mice. PLoS One (2012) 7(5):e37286. doi: 10.1371/journal.pone.0037286 22615967 PMC3352873

[48] Shankar SP Wilson MS Divietro JA Mentink-Kane MM Xie Z Wynn TA. RGS16 attenuates pulmonary Th2/Th17 inflammatory responses. J Immunol (2012) 188(12):6347–56. doi: 10.4049/jimmunol.1103781 PMC3522182 22593615

[49] Mishra NC Rir-Sima-Ah J Grotendorst GR Langley RJ Singh SP Gundavarapu S. Inhalation of sulfur mustard causes long-term T cell-dependent inflammation: possible role of Th17 cells in chronic lung pathology. Int Immunopharmacol (2012) 13(1):101–8. doi: 10.1016/j.intimp.2012.03.010 PMC3340497 22465472

[50] Dong Z Tai W Yang Y Zhang T Li Y Chai Y. The role of all-trans retinoic acid in bleomycin-induced pulmonary fibrosis in mice. Exp Lung Res (2012) 38(2):82–9. doi: 10.3109/01902148.2011.646052 22250783

[51] Yoshizaki A Yanaba K Iwata Y Komura K Ogawa A Akiyama Y. Cell adhesion molecules regulate fibrotic process *via* Th1/Th2/Th17 cell balance in a bleomycin-induced scleroderma model. J Immunol (2010) 185(4):2502–15. doi: 10.4049/jimmunol.0901778 PMC3733122 20624949

[52] Braun RK Martin A Shah S Iwashima M Medina M Byrne K. Inhibition of bleomycin-induced pulmonary fibrosis through pre-treatment with collagen type V. J Heart Lung Transplant (2010) 29(8):873–80. doi: 10.1016/j.healun.2010.03.012 20471860

[53] Wilson MS Madala SK Ramalingam TR Gochuico BR Rosas IO Cheever AW. Bleomycin and IL-1beta-mediated pulmonary fibrosis is IL-17A dependent. J Exp Med (2010) 207(3):535–52. doi: 10.1084/jem.20092121 PMC2839145 20176803

[54] Cipolla E Fisher AJ Gu H Mickler EA Agarwal M Wilke CA. IL-17A deficiency mitigates bleomycin-induced complement activation during lung fibrosis. FASEB J (2017) 31(12):5543–56. doi: 10.1096/fj.201700289R PMC5690386 28821630

[55] Paun A Bergeron ME Haston CK. The Th1/Th17 balance dictates the fibrosis response in murine radiation-induced lung disease. Sci Rep (2017) 7(1):11586. doi: 10.1038/s41598-017-11656-5 28912510 PMC5599556

[56] Braun NA Celada LJ Herazo-Maya JD Abraham S Shaginurova G Sevin CM. Blockade of the programmed death-1 pathway restores sarcoidosis CD4(+) T-cell proliferative capacity. Am J Respir Crit Care Med (2014) 190(5):560–71. doi: 10.1164/rccm.201401-0188OC PMC4214083 25073001

[57] Kuo IY Yang YE Yang PS Tsai YJ Tzeng HT Cheng HC. Converged Rab37/IL-6 trafficking and STAT3/PD-1 transcription axes elicit an immunosuppressive lung tumor microenvironment. Theranostics (2021) 11(14):7029–44. doi: 10.7150/thno.60040 PMC8171097 34093869

[58] Kondo K Shaim H Thompson PA Burger JA Keating M Estrov Z. Ibrutinib modulates the immunosuppressive CLL microenvironment through STAT3-mediated suppression of regulatory b-cell function and inhibition of the PD-1/PD-L1 pathway. Leukemia (2018) 32(4):960–70. doi: 10.1038/leu.2017.304 PMC6128536 28972595

[59] Krasilnikov M Ivanov VN Dong J Ronai Z. ERK and PI3K negatively regulate STAT-transcriptional activities in human melanoma cells: implications towards sensitization to apoptosis. Oncogene (2003) 22(26):4092–101. doi: 10.1038/sj.onc.1206598 12821943

[60] Celada LJ Rotsinger JE Young A Shaginurova G Shelton D Hawkins C. Programmed death-1 inhibition of phosphatidylinositol 3-Kinase/AKT/Mechanistic target of rapamycin signaling impairs sarcoidosis CD4(+) T cell proliferation. Am J Respir Cell Mol Biol (2017) 56(1):74–82. doi: 10.1165/rcmb.2016-0037OC 27564547 PMC5248958

[61] Knochelmann HM Dwyer CJ Bailey SR Amaya SM Elston DM Mazza-Mccrann JM. When worlds collide: Th17 and treg cells in cancer and autoimmunity. Cell Mol Immunol (2018) 15(5):458–69. doi: 10.1038/s41423-018-0004-4 PMC6068176 29563615

[62] Koch MA Tucker-Heard G Perdue NR Killebrew JR Urdahl KB Campbell DJ. The transcription factor T-bet controls regulatory T cell homeostasis and function during type 1 inflammation. Nat Immunol (2009) 10(6):595–602. doi: 10.1038/ni.1731 19412181 PMC2712126

[63] Yang XO Nurieva R Martinez GJ Kang HS Chung Y Pappu BP. Molecular antagonism and plasticity of regulatory and inflammatory T cell programs. Immunity (2008) 29(1):44–56. doi: 10.1016/j.immuni.2008.05.007 18585065 PMC2630532

[64] Liu SS Liu C Lv XX Cui B Yan J Li YX. The chemokine CCL1 triggers an AMFR-SPRY1 pathway that promotes differentiation of lung fibroblasts into myofibroblasts and drives pulmonary fibrosis. Immunity (2021) 54(9):2042–56.e8. doi: 10.1016/j.immuni.2021.06.008 34407391

[65] Andugulapati SB Gourishetti K Tirunavalli SK Shaikh TB Sistla R. Biochanin-a ameliorates pulmonary fibrosis by suppressing the TGF-β mediated EMT, myofibroblasts differentiation and collagen deposition in *in vitro* and *in vivo* systems. Phytomedicine (2020) 78:153298. doi: 10.1016/j.phymed.2020.153298 32781391 PMC7395646

[66] Yan J Yao Y Yan S Gao R Lu W He W. Chiral protein supraparticles for tumor suppression and synergistic immunotherapy: An enabling strategy for bioactive supramolecular chirality construction. . Nano Lett (2020) 20(8):5844–52. doi: 10.1021/acs.nanolett.0c01757 32589431

[67] Yan J He W Yan S Niu F Liu T Ma B. Self-assembled peptide-lanthanide nanoclusters for safe tumor therapy: Overcoming and utilizing biological barriers to peptide drug delivery. ACS Nano (2018) 12(2):2017–26. doi: 10.1021/acsnano.8b00081 29376322

[68] He W Yan J Jiang W Li S Qu Y Niu F. Peptide-induced self-assembly of therapeutics into a well-defined nanoshell with tumor-triggered shape and charge switch. Chem Mater (2018) 30(20):7034–46. doi: 10.1021/acs.chemmater.8b02572 PMC7518337 32982042

[69] Bian Z Yan J Wang S Li Y Guo Y Ma B. Awakening p53 *in vivo* by d-peptides-functionalized ultra-small nanoparticles: Overcoming biological barriers to d-peptide drug delivery. Theranostics (2018) 8(19):5320–35. doi: 10.7150/thno.27165 PMC6276095 30555549

[70] Wei J Jin L Liu M Hou P He W-X. A dimethylbromobenzene-cysteine stapled peptide dual inhibitor of the p53-MDM2/MDMX interactions. Hepatoma Res (2019) 5:5. doi: 10.20517/2394-5079.2018.97

[71] Shetty SK Bhandary YP Marudamuthu AS Abernathy D Velusamy T Starcher B. Regulation of airway and alveolar epithelial cell apoptosis by p53-induced plasminogen activator inhibitor-1 during cigarette smoke exposure injury. Am J Respir Cell Mol Biol (2012) 47(4):474–83. doi: 10.1165/rcmb.2011-0390OC PMC3488631 22592924

[72] Bhandary YP Shetty SK Marudamuthu AS Gyetko MR Idell S Gharaee-Kermani M. Regulation of alveolar epithelial cell apoptosis and pulmonary fibrosis by coordinate expression of components of the fibrinolytic system. Am J Physiol Lung Cell Mol Physiol (2012) 302(5):L463–73. doi: 10.1152/ajplung.00099.2011 PMC3311514 22140072

[73] Shetty SK Tiwari N Marudamuthu AS Puthusseri B Bhandary YP Fu J. p53 and miR-34a feedback promotes lung epithelial injury and pulmonary fibrosis. Am J Pathol (2017) 187(5):1016–34. doi: 10.1016/j.ajpath.2016.12.020 PMC5417006 28273432

[74] Cisneros J Hagood J Checa M Ortiz-Quintero B Negreros M Herrera I. Hypermethylation-mediated silencing of p14(ARF) in fibroblasts from idiopathic pulmonary fibrosis. Am J Physiol Lung Cell Mol Physiol (2012) 303(4):L295–303. doi: 10.1152/ajplung.00332.2011 PMC6189747 22707614

[75] Qu J Yang SZ Zhu Y Guo T Thannickal VJ Zhou Y. Targeting mechanosensitive MDM4 promotes lung fibrosis resolution in aged mice. J Exp Med (2021) 218(5):e20202033. doi: 10.1084/jem.20202033 33688918 PMC7953267

[76] Cortez MA Ivan C Valdecanas D Wang X Peltier HJ Ye Y. PDL1 regulation by p53 *via* miR-34. J Natl Cancer Inst (2016) 108(1):djv303. doi: 10.1093/jnci/djv303 26577528 PMC4862407

[77] Chen L Gibbons DL Goswami S Cortez MA Ahn YH Byers LA. Metastasis is regulated *via* microRNA-200/ZEB1 axis control of tumour cell PD-L1 expression and intratumoral immunosuppression. Nat Commun (2014) 5:5241. doi: 10.1038/ncomms6241 25348003 PMC4212319

[78] Costa C Indovina P Mattioli E Forte IM Iannuzzi CA Luzzi L. P53-regulated miR-320a targets PDL1 and is downregulated in malignant mesothelioma. Cell Death Dis (2020) 11(9):748. doi: 10.1038/s41419-020-02940-w 32929059 PMC7490273

[79] Antoniades HN Galanopoulos T Neville-Golden J Kiritsy CP Lynch SE. p53 expression during normal tissue regeneration in response to acute cutaneous injury in swine. J Clin Invest (1994) 93(5):2206–14. doi: 10.1172/jci117217 PMC294365 8182152

[80] Desmoulière A Redard M Darby I Gabbiani G. Apoptosis mediates the decrease in cellularity during the transition between granulation tissue and scar. Am J Pathol (1995) 146(1):56–66. 7856739 PMC1870783

[81] Mohan N Hosain S Zhao J Shen Y Luo X Jiang J. Atezolizumab potentiates tcell-mediated cytotoxicity and coordinates with FAK to suppress cell invasion and motility in PD-L1(+) triple negative breast cancer cells. Oncoimmunology (2019) 8(9):e1624128. doi: 10.1080/2162402x.2019.1624128 31428520 PMC6685513

[82] Flanders KC. Smad3 as a mediator of the fibrotic response. Int J Exp Pathol (2004) 85(2):47–64. doi: 10.1111/j.0959-9673.2004.00377.x 15154911 PMC2517464

[83] Yu J Qin B Moyer AM Nowsheen S Tu X Dong H. Regulation of sister chromatid cohesion by nuclear PD-L1. Cell Res (2020) 30(7):590–601. doi: 10.1038/s41422-020-0315-8 32350394 PMC7343880

[84] Gao Y Nihira NT Bu X Chu C Zhang J Kolodziejczyk A. Acetylation-dependent regulation of PD-L1 nuclear translocation dictates the efficacy of anti-PD-1 immunotherapy. Nat Cell Biol (2020) 22(9):1064–75. doi: 10.1038/s41556-020-0562-4 PMC7484128 32839551

[85] Yu W Hua Y Qiu H Hao J Zou K Li Z. PD-L1 promotes tumor growth and progression by activating WIP and β-catenin signaling pathways and predicts poor prognosis in lung cancer. Cell Death Dis (2020) 11(7):506. doi: 10.1038/s41419-020-2701-z 32632098 PMC7338457

[86] Chilosi M Poletti V Zamò A Lestani M Montagna L Piccoli P. Aberrant wnt/beta-catenin pathway activation in idiopathic pulmonary fibrosis. Am J Pathol (2003) 162(5):1495–502. doi: 10.1016/s0002-9440(10)64282-4 PMC1851206 12707032

[87] Han J Hou W Goldstein LA Stolz DB Watkins SC Rabinowich H. A complex between Atg7 and caspase-9: A NOVEL MECHANISM OF CROSS-REGULATION BETWEEN AUTOPHAGY AND APOPTOSIS. J Biol Chem (2014) 289(10):6485–97. doi: 10.1074/jbc.M113.536854 PMC3945314 24362031

[88] Jiang A Liu N Bai S Wang J Gao H Zheng X. Identification and validation of an autophagy-related long non-coding RNA signature as a prognostic biomarker for patients with lung adenocarcinoma. J Thorac Dis (2021) 13(2):720–34. doi: 10.21037/jtd-20-2803 PMC7947511 33717544

[89] Klionsky DJ. Autophagy: from phenomenology to molecular understanding in less than a decade. Nat Rev Mol Cell Biol (2007) 8(11):931–7. doi: 10.1038/nrm2245 17712358

[90] Levine B Mizushima N Virgin HW. Autophagy in immunity and inflammation. Nat (2011) 469(7330):323–35. doi: 10.1038/nature09782 PMC3131688 21248839

[91] Sharma P Alizadeh J Juarez M Samali A Halayko AJ Kenyon NJ. Apoptosis, the unfolded protein response, and lung function in idiopathic pulmonary fibrosis. Cells (2021) 10(7):1642. doi: 10.3390/cells10071642 34209019 PMC8307368

[92] Li X Zhao F Wang A Cheng P Chen H. Role and mechanisms of autophagy in lung metabolism and repair. Cell Mol Life Sci (2021) 78(12):5051–68. doi: 10.1007/s00018-021-03841-7 PMC11072280 33864479

[93] Annangi B Lu Z Bruniaux J Ridoux A Da Silva VM Vantelon D. Macrophage autophagy protects mice from cerium oxide nanoparticle-induced lung fibrosis. Part Fibre Toxicol (2021) 18(1):6. doi: 10.1186/s12989-021-00398-y 33526046 PMC7852145

[94] Baek AR Hong J Song KS Jang AS Kim DJ Chin SS. Spermidine attenuates bleomycin-induced lung fibrosis by inducing autophagy and inhibiting endoplasmic reticulum stress (ERS)-induced cell death in mice. Exp Mol Med (2020) 52(12):2034–45. doi: 10.1038/s12276-020-00545-z PMC8080799 33318630

[95] Sosulski ML Gongora R Danchuk S Dong C Luo F Sanchez CG. Deregulation of selective autophagy during aging and pulmonary fibrosis: the role of TGFβ1. Aging Cell (2015) 14(5):774–83. doi: 10.1111/acel.12357 PMC4568965 26059457

[96] Cabrera S Maciel M Herrera I Nava T Vergara F Gaxiola M. Essential role for the ATG4B protease and autophagy in bleomycin-induced pulmonary fibrosis. Autophagy (2015) 11(4):670–84. doi: 10.1080/15548627.2015.1034409 PMC4502665 25906080

[97] Wang K Zhang T Lei Y Li X Jiang J Lan J. Identification of ANXA2 (annexin A2) as a specific bleomycin target to induce pulmonary fibrosis by impeding TFEB-mediated autophagic flux. Autophagy (2018) 14(2):269–82. doi: 10.1080/15548627.2017.1409405 PMC5902212 29172997

[98] Araya J Kojima J Takasaka N Ito S Fujii S Hara H. Insufficient autophagy in idiopathic pulmonary fibrosis. Am J Physiol Lung Cell Mol Physiol (2013) 304(1):L56–69. doi: 10.1152/ajplung.00213.2012 23087019

[99] Lawrence J Nho R. The role of the mammalian target of rapamycin (mTOR) in pulmonary fibrosis. Int J Mol Sci (2018) 19(3):778. doi: 10.3390/ijms19030778 29518028 PMC5877639

[100] Conte E Fruciano M Fagone E Gili E Caraci F Iemmolo M. Inhibition of PI3K prevents the proliferation and differentiation of human lung fibroblasts into myofibroblasts: the role of class I P110 isoforms. PLoS One (2011) 6(10):e24663. doi: 10.1371/journal.pone.0024663 21984893 PMC3184941

[101] Sun Y Zhang Y Chi P. Pirfenidone suppresses TGF−β1−induced human intestinal fibroblasts activities by regulating proliferation and apoptosis *via* the inhibition of the smad and PI3K/AKT signaling pathway. Mol Med Rep (2018) 18(4):3907–13. doi: 10.3892/mmr.2018.9423 PMC6131636 30152848

[102] Yu JZ Ying Y Liu Y Sun CB Dai C Zhao S. Antifibrotic action of yifei sanjie formula enhanced autophagy *via* PI3K-AKT-mTOR signaling pathway in mouse model of pulmonary fibrosis. BioMed Pharmacother (2019) 118:109293. doi: 10.1016/j.biopha.2019.109293 31401393

[103] Clark CA Gupta HB Sareddy G Pandeswara S Lao S Yuan B. Tumor-intrinsic PD-L1 signals regulate cell growth, pathogenesis, and autophagy in ovarian cancer and melanoma. Cancer Res (2016) 76(23):6964–74. doi: 10.1158/0008-5472.Can-16-0258 PMC5228566 27671674

[104] Wang X Wu WKK Gao J Li Z Dong B Lin X. Autophagy inhibition enhances PD-L1 expression in gastric cancer. J Exp Clin Cancer Res (2019) 38(1):140. doi: 10.1186/s13046-019-1148-5 30925913 PMC6440013

[105] Crunkhorn S. Inflammatory disease: T cell-targeted antibody reverses fibrosis. Nat Rev Drug Discovery (2016) 15(8):530–1. doi: 10.1038/nrd.2016.144 27444224

[106] Hou Z Ye Q Qiu M Hao Y Han J Zeng H. Increased activated regulatory T cells proportion correlate with the severity of idiopathic pulmonary fibrosis. Respir Res (2017) 18(1):170. doi: 10.1186/s12931-017-0653-3 28886713 PMC5591546

[107] Moore MW Herzog EL. Regulatory T cells in idiopathic pulmonary fibrosis: Too much of a good thing? Am J Pathol (2016) 186(8):1978–81. doi: 10.1016/j.ajpath.2016.06.002 PMC4973652 27344432

[108] Jaiswal S Jamieson CH Pang WW Park CY Chao MP Majeti R. CD47 is upregulated on circulating hematopoietic stem cells and leukemia cells to avoid phagocytosis. Cell (2009) 138(2):271–85. doi: 10.1016/j.cell.2009.05.046 PMC2775564 19632178

[109] Majeti R Chao MP Alizadeh AA Pang WW Jaiswal S Gibbs KD Jr.. CD47 is an adverse prognostic factor and therapeutic antibody target on human acute myeloid leukemia stem cells. Cell (2009) 138(2):286–99. doi: 10.1016/j.cell.2009.05.045 PMC2726837 19632179

[110] Fernandez IE Greiffo FR Frankenberger M Bandres J Heinzelmann K Neurohr C. Peripheral blood myeloid-derived suppressor cells reflect disease status in idiopathic pulmonary fibrosis. Eur Respir J (2016) 48(4):1171–83. doi: 10.1183/13993003.01826-2015 27587556

[111] Wynn TA Ramalingam TR. Mechanisms of fibrosis: therapeutic translation for fibrotic disease. Nat Med (2012) 18(7):1028–40. doi: 10.1038/nm.2807 PMC3405917 22772564

[112] Blackwell TS Tager AM Borok Z Moore BB Schwartz DA Anstrom KJ. Future directions in idiopathic pulmonary fibrosis research. an NHLBI workshop report. Am J Respir Crit Care Med (2014) 189(2):214–22. doi: 10.1164/rccm.201306-1141WS PMC3983890 24160862

[113] Gilani SR Vuga LJ Lindell KO Gibson KF Xue J Kaminski N. CD28 down-regulation on circulating CD4 T-cells is associated with poor prognoses of patients with idiopathic pulmonary fibrosis. PLoS One (2010) 5(1):e8959. doi: 10.1371/journal.pone.0008959 20126467 PMC2813297

[114] Herazo-Maya JD Noth I Duncan SR Kim S Ma SF Tseng GC. Peripheral blood mononuclear cell gene expression profiles predict poor outcome in idiopathic pulmonary fibrosis. Sci Transl Med (2013) 5(205):205ra136. doi: 10.1126/scitranslmed.3005964 PMC4175518 24089408

[115] Meijers RW Litjens NH De Wit EA Langerak AW van der Spek A Baan CC. Cytomegalovirus contributes partly to uraemia-associated premature immunological ageing of the T cell compartment. Clin Exp Immunol (2013) 174(3):424–32. doi: 10.1111/cei.12188 PMC3826308 23962178

[116] Vallejo AN. CD28 extinction in human T cells: altered functions and the program of T-cell senescence. Immunol Rev (2005) 205:158–69. doi: 10.1111/j.0105-2896.2005.00256.x 15882352

[117] Chen X Huang J Wu J Hao J Fu B Wang Y. Human mesenchymal stem cells. Cell Prolif (2022) 55(4):e13141. doi: 10.1111/cpr.13141 34936710 PMC9055891

[118] Yan J Zhang L Li L He W Liu W. Developmentally engineered bio-assemblies releasing neurotrophic exosomes guide *in situ* neuroplasticity following spinal cord injury. Materials Today Bio (2022) 16:100406. doi: 10.1016/j.mtbio.2022.100406 PMC9440432 36065352

[119] Abumaree M Jumah M Pace RA Kalionis B. Immunosuppressive properties of mesenchymal stem cells. Stem Cell Rev Rep (2012) 8(2):375–92. doi: 10.1007/s12015-011-9312-0 21892603

[120] Zhou T Yuan Z Weng J Pei D Du X He C. Challenges and advances in clinical applications of mesenchymal stromal cells. J Hematol Oncol (2021) 14(1):24. doi: 10.1186/s13045-021-01037-x 33579329 PMC7880217

[121] Krampera M. Mesenchymal stromal cell 'licensing': A multistep process. Leukemia (2011) 25(9):1408–14. doi: 10.1038/leu.2011.108 21617697

[122] Shi Y Su J Roberts AI Shou P Rabson AB Ren G. How mesenchymal stem cells interact with tissue immune responses. Trends Immunol (2012) 33(3):136–43. doi: 10.1016/j.it.2011.11.004 PMC3412175 22227317

[123] Parry RV Chemnitz JM Frauwirth KA Lanfranco AR Braunstein I Kobayashi SV. CTLA-4 and PD-1 receptors inhibit T-cell activation by distinct mechanisms. Mol Cell Biol (2005) 25(21):9543–53. doi: 10.1128/mcb.25.21.9543-9553.2005 PMC1265804 16227604

[124] Qin W Zou J Huang Y Liu C Kang Y Han H. Pirfenidone facilitates immune infiltration and enhances the antitumor efficacy of PD-L1 blockade in mice. Oncoimmunology (2020) 9(1):1824631. doi: 10.1080/2162402x.2020.1824631 33457101 PMC7781712

[125] Yang S Liu P Jiang Y Wang Z Dai H Wang C. Therapeutic applications of mesenchymal stem cells in idiopathic pulmonary fibrosis. Front Cell Dev Biol (2021) 9:639657. doi: 10.3389/fcell.2021.639657 33768094 PMC7985078

[126] Papiris SA Kollintza A Karatza M Manali ED Sotiropoulou C Milic-Emili J. CD8+ T lymphocytes in bronchoalveolar lavage in idiopathic pulmonary fibrosis. J Inflammation (Lond) (2007) 4:14. doi: 10.1186/1476-9255-4-14 PMC1906752 17578573

[127] Daniil Z Kitsanta P Kapotsis G Mathioudaki M Kollintza A Karatza M. Papiris. CD8+ T lymphocytes in lung tissue from patients with idiopathic pulmonary fibrosis. Respir Res (2005) 6(1):81. doi: 10.1186/1465-9921-6-81 16042790 PMC1199622

[128] Duitman J Ende TVD Spek CA. Immune checkpoints as promising targets for the treatment of idiopathic pulmonary fibrosis? J Clin Med (2019) 8(10):1547. doi: 10.3390/jcm8101547 31561518 PMC6833050

[129] Wang W Wang Q Xu C Li Z Song Z Zhang Y. Chinese Expert consensus on the multidisciplinary management of pneumonitis associated with immune checkpoint inhibitor. Thorac Cancer (2022) 10:14693. doi: 10.1111/1759-7714.14693 PMC9715776 36268845

[130] Hunter CA Jones SA. IL-6 as a keystone cytokine in health and disease. Nat Immunol (2015) 16(5):448–57. doi: 10.1038/ni.3153 25898198

[131] Le TT Karmouty-Quintana H Melicoff E Le TT Weng T Chen NY. Blockade of IL-6 trans signaling attenuates pulmonary fibrosis. J Immunol (2014) 193(7):3755–68. doi: 10.4049/jimmunol.1302470 PMC4169999 25172494

[132] Liu J Wang L Zhao F Tseng S Narayanan C Shura L. Pre-clinical development of a humanized anti-CD47 antibody with anti-cancer therapeutic potential. PLoS One (2015) 10(9):e0137345. doi: 10.1371/journal.pone.0137345 26390038 PMC4577081

[133] Willingham SB Volkmer JP Gentles AJ Sahoo D Dalerba P Mitra SS. The CD47-signal regulatory protein alpha (SIRPa) interaction is a therapeutic target for human solid tumors. Proc Natl Acad Sci U.S.A. (2012) 109(17):6662–7. doi: 10.1073/pnas.1121623109 PMC3340046 22451913

[134] Gordon SR Maute RL Dulken BW Hutter G George BM Mccracken MN. PD-1 expression by tumour-associated macrophages inhibits phagocytosis and tumour immunity. Nature (2017) 545(7655):495–9. doi: 10.1038/nature22396 PMC5931375 28514441

[135] Hsieh RC Krishnan S Wu RC Boda AR Liu A Winkler M. ATR-mediated CD47 and PD-L1 up-regulation restricts radiotherapy-induced immune priming and abscopal responses in colorectal cancer. Sci Immunol (2022) 7(72):eabl9330. doi: 10.1126/sciimmunol.abl9330 35687697 PMC9373855

[136] Chen SH Dominik PK Stanfield J Ding S Yang W Kurd N. Dual checkpoint blockade of CD47 and PD-L1 using an affinity-tuned bispecific antibody maximizes antitumor immunity. J Immunother Cancer (2021) 9(10):e003464. doi: 10.1136/jitc-2021-003464 34599020 PMC8488710

[137] Wang Y Ni H Zhou S He K Gao Y Wu W. Tumor-selective blockade of CD47 signaling with a CD47/PD-L1 bispecific antibody for enhanced anti-tumor activity and limited toxicity. Cancer Immunol Immunother (2021) 70(2):365–76. doi: 10.1007/s00262-020-02679-5 PMC10991320 32761423

[138] Abu Hejleh T Furqan M Ballas Z Clamon G. The clinical significance of soluble PD-1 and PD-L1 in lung cancer. Crit Rev Oncol Hematol (2019) 143:148–52. doi: 10.1016/j.critrevonc.2019.08.009 31675543

[139] Gu D Ao X Yang Y Chen Z Xu X. Soluble immune checkpoints in cancer: production, function and biological significance. J Immunother Cancer (2018) 6(1):132. doi: 10.1186/s40425-018-0449-0 30482248 PMC6260693

[140] Sorensen SF Demuth C Weber B Sorensen BS Meldgaard P. Increase in soluble PD-1 is associated with prolonged survival in patients with advanced EGFR-mutated non-small cell lung cancer treated with erlotinib. Lung Cancer (2016) 100:77–84. doi: 10.1016/j.lungcan.2016.08.001 27597284

[141] Okuma Y Wakui H Utsumi H Sagawa Y Hosomi Y Kuwano K. Soluble programmed cell death ligand 1 as a novel biomarker for nivolumab therapy for non-small-cell lung cancer. Clin Lung Cancer (2018) 19(5):410–7.e1. doi: 10.1016/j.cllc.2018.04.014 29859759

[142] Singh A Chakraborty S Wong SW Hefner NA Stuart A Qadir AS. Nanoparticle targeting of *de novo* profibrotic macrophages mitigates lung fibrosis. Proc Natl Acad Sci U.S.A. (2022) 119(15):e2121098119. doi: 10.1073/pnas.2121098119 35377803 PMC9169714

[143] Mccubbrey AL Barthel L Mohning MP Redente EF Mould KJ Thomas SM. Deletion of c-FLIP from CD11b(hi) macrophages prevents development of bleomycin-induced lung fibrosis. Am J Respir Cell Mol Biol (2018) 58(1):66–78. doi: 10.1165/rcmb.2017-0154OC 28850249 PMC5941310

[144] Misharin AV Morales-Nebreda L Reyfman PA Cuda CM Walter JM Mcquattie-Pimentel AC. Monocyte-derived alveolar macrophages drive lung fibrosis and persist in the lung over the life span. J Exp Med (2017) 214(8):2387–404. doi: 10.1084/jem.20162152 PMC5551573 28694385

[145] Aran D Looney AP Liu L Wu E Fong V Hsu A. Reference-based analysis of lung single-cell sequencing reveals a transitional profibrotic macrophage. . Nat Immunol (2019) 20(2):163–72. doi: 10.1038/s41590-018-0276-y PMC6340744 30643263

[146] Hartley GP Chow L Ammons DT Wheat WH Dow SW. Programmed cell death ligand 1 (PD-L1) signaling regulates macrophage proliferation and activation. Cancer Immunol Res (2018) 6(10):1260–73. doi: 10.1158/2326-6066.Cir-17-0537 30012633

[147] Jiang A Wang J Liu N Zheng X Li Y Ma Y. Integration of single-cell RNA sequencing and bulk RNA sequencing data to establish and validate a prognostic model for patients with lung adenocarcinoma. Front Genet (2022) 13:833797. doi: 10.3389/fgene.2022.833797 35154287 PMC8829512

[148] Rahman M Wang ZY Li JX Xu HW Wang R Wu Q. Single-cell RNA sequencing reveals the interaction of injected ADSCs with lung-originated cells in mouse pulmonary fibrosis. Stem Cells Int (2022) 2022:9483166. doi: 10.1155/2022/9483166 35450342 PMC9017459

[149] Heinzelmann K Hu Q Hu Y Dobrinskikh E Ansari M Melo-Narváez MC. Single-cell RNA sequencing identifies G-protein coupled receptor 87 as a basal cell marker expressed in distal honeycomb cysts in idiopathic pulmonary fibrosis. Eur Respir J (2022) 59(6):2102373. doi: 10.1183/13993003.02373-2021 35604813 PMC9203838

[150] Valenzi E Tabib T Papazoglou A Sembrat J Trejo Bittar HE Rojas M. Disparate interferon signaling and shared aberrant basaloid cells in single-cell profiling of idiopathic pulmonary fibrosis and systemic sclerosis-associated interstitial lung disease. Front Immunol (2021) 12:595811. doi: 10.3389/fimmu.2021.595811 33859634 PMC8042271

[151] Nakahara Y Hashimoto N Sakamoto K Enomoto A Adams TS Yokoi T. Fibroblasts positive for meflin have anti-fibrotic properties in pulmonary fibrosis. Eur Respir J (2021) 58(6):2003397. doi: 10.1183/13993003.03397-2020 34049947

[152] Fastrès A Pirottin D Fievez L Tutunaru AC Bolen G Merveille AC. Identification of pro-fibrotic macrophage populations by single-cell transcriptomic analysis in West highland white terriers affected with canine idiopathic pulmonary fibrosis. Front Immunol (2020) 11:611749. doi: 10.3389/fimmu.2020.611749 33384697 PMC7770158

[153] Lv T Jiang K Wang J Tang N Dai H Wang C. Single-cell RNA sequencing profiling of the effects of aging on alveolar stem cells. Sci China Life Sci (2019) 62(8):1028–37. doi: 10.1007/s11427-019-9583-9 31321669

[154] Xu Y Mizuno T Sridharan A Du Y Guo M Tang J. Single-cell RNA sequencing identifies diverse roles of epithelial cells in idiopathic pulmonary fibrosis. . JCI Insight (2016) 1(20):e90558. doi: 10.1172/jci.insight.90558 27942595 PMC5135277

[155] Reyfman PA Walter JM Joshi N Anekalla KR Mcquattie-Pimentel AC Chiu S. Single-cell transcriptomic analysis of human lung provides insights into the pathobiology of pulmonary fibrosis. Am J Respir Crit Care Med (2019) 199(12):1517–36. doi: 10.1164/rccm.201712-2410OC PMC6580683 30554520

[156] Adams Taylor S Schupp JC Poli S Ayaub EA Neumark N Ahangari F. Single-cell RNA-seq reveals ectopic and aberrant lung-resident cell populations in idiopathic pulmonary fibrosis. Sci Adv (2020) 6(28):eaba1983. doi: 10.1126/sciadv.aba1983 32832599 PMC7439502

[157] Habermann Arun C Gutierrez AJ Bui LT Yahn SL Winters NI Calvi CL. Single-cell RNA sequencing reveals profibrotic roles of distinct epithelial and mesenchymal lineages in pulmonary fibrosis. Sci Adv (2020) 6(28):eaba1972. doi: 10.1126/sciadv.aba1972 32832598 PMC7439444

[158] Wang Y Li X Zheng D Chen Y Zhang Z Yang Z. Selective degradation of PD-L1 in cancer cells by enzyme-instructed self-assembly. Adv Funct Materials (2021) 31(45):2102505. doi: 10.1002/adfm.202102505

[159] Cotton AD Nguyen DP Gramespacher JA Seiple IB Wells JA. Development of antibody-based PROTACs for the degradation of the cell-surface immune checkpoint protein PD-L1. J Am Chem Soc (2021) 143(2):593–8. doi: 10.1021/jacs.0c10008 PMC8154509 33395526

[160] Yan S Yan J Liu D Li X Kang Q You W. A nano-predator of pathological MDMX construct by clearable supramolecular gold(I)-thiol-peptide complexes achieves safe and potent anti-tumor activity. Theranostics (2021) 11(14):6833–46. doi: 10.7150/thno.59020 PMC8171083 34093856

[161] Yang W Liu W Li X Yan J He W. Turning chiral peptides into a racemic supraparticle to induce the self-degradation of MDM2. J Adv Res (2022) S2090–1232(22):00121–7. doi: 10.1016/j.jare.2022.05.009 PMC10006529 35667548

[162] Yan J Ji F Yan S You W Ma F Li F. A general-purpose nanohybrid fabricated by polymeric Au(I)-peptide precursor to wake the function of peptide therapeutics. Theranostics (2020) 10(19):8513–27. doi: 10.7150/thno.47243 PMC7392018 32754260

[163] Yan J Zheng X You W He W Xu G-K. A bionic-homodimerization strategy for optimizing modulators of protein–protein interactions: From statistical mechanics theory to potential clinical translation. Adv Sci (2022) 9(11):2105179. doi: 10.1002/advs.202105179 PMC9008432 35166067

[164] She J Li Y Yan S Yan Y Liu D Li S. *De novo* supraparticle construction by a self-assembled janus cyclopeptide to tame hydrophilic microRNA and hydrophobic molecule for anti-tumor cocktail therapy and augmented immunity. Chem Eng J (2020) 401:126080. doi: 10.1016/j.cej.2020.126080

[165] Yan J He W Yan S Niu F Liu T Ma B. Self-assembled peptide–lanthanide nanoclusters for safe tumor therapy: Overcoming and utilizing biological barriers to peptide drug delivery. ACS Nano (2018) 12(2):2017–26. doi: 10.1021/acsnano.8b00081 29376322

[166] Tran TH Phuong Tran TT. Targeting the PD-1/PD-L1 axis for cancer treatment: A review on nanotechnology. R Soc Open Sci (2022) 9(4):211991. doi: 10.1098/rsos.211991 35425626 PMC9006034

[167] Niu F Yan J Ma B Li S Shao Y He P. Lanthanide-doped nanoparticles conjugated with an anti-CD33 antibody and a p53-activating peptide for acute myeloid leukemia therapy. Biomaterials (2018) 167:132–42. doi: 10.1016/j.biomaterials.2018.03.025 PMC5889738 29571049

